# Infection Susceptibility in Gastric Intrinsic Factor (Vitamin B_12_)-Defective Mice Is Subject to Maternal Influences

**DOI:** 10.1128/mBio.00830-16

**Published:** 2016-06-21

**Authors:** Lynda Mottram, Anneliese O. Speak, Reza M. Selek, Emma L. Cambridge, Zoe McIntyre, Leanne Kane, Subhankar Mukhopadhyay, Carolyn Grove, Amy Colin, Cordelia Brandt, Maria A. Duque-Correa, Jessica Forbester, Tu Anh Pham Nguyen, Christine Hale, George S. Vasilliou, Mark J. Arends, Brendan W. Wren, Gordon Dougan, Simon Clare

**Affiliations:** a Wellcome Trust Sanger Institute, Wellcome Trust Genome Campus, Hinxton, United Kingdom; b Wellcome Trust Genome Campus, European Bioinformatics Institute, Cheminformatics and Metabolism, Hinxton, United Kingdom; c Department of Biochemistry and Cambridge Systems Biology Centre, University of Cambridge, Cambridge, United Kingdom; d Division of Pathology, University Of Edinburgh, Edinburgh, United Kingdom; e Department of Pathogen Molecular Biology, London School of Hygiene and Tropical Medicine, London, United Kingdom

## Abstract

Mice harboring a mutation in the gene encoding gastric intrinsic factor (Gif), a protein essential for the absorption of vitamin B_12_/cobalamin (Cbl), have potential as a model to explore the role of vitamins in infection. The levels of Cbl in the blood of *Gif*^tm1a/tm1a^ mutant mice were influenced by the maternal genotype, with offspring born to heterozygous (high Cbl, F_1_) mothers exhibiting a significantly higher serum Cbl level than those born to homozygous (low Cbl, F_2_) equivalents. Low Cbl levels correlated with susceptibility to an infectious challenge with *Salmonella enterica* serovar Typhimurium or *Citrobacter rodentium*, and this susceptibility phenotype was moderated by Cbl administration. Transcriptional and metabolic profiling revealed that Cbl deficient mice exhibited a bioenergetic shift similar to a metabolic phenomenon commonly found in cancerous cells under hypoxic conditions known as the Warburg effect, with this metabolic effect being exacerbated further by infection. Our findings demonstrate a role for Cbl in bacterial infection, with potential general relevance to dietary deficiency and infection susceptibility.

## IMPORTANCE

Malnutrition continues to be a major public health problem in countries with weak infrastructures. In communities with a high prevalence of poor diet, malnourishment and infectious disease can impact vulnerable individuals such as pregnant women and children. Here, we describe a highly flexible murine model for monitoring maternal and environmental influences of vitamin B_12_ metabolism. We also demonstrate the potential importance of vitamin B_12_ in controlling susceptibility to bacterial pathogens such as *C. rodentium* and *S*. Typhimurium. We postulate that this model, along with similarly vitamin deficient mice, could be used to further explore the mechanisms associated with micronutrients and susceptibility to diseases, thereby increasing our understanding of disease in the malnourished.

## INTRODUCTION

Clinical and epidemiological studies have demonstrated an association of malnutrition, dietary deficiency, and infection (1, 2). Infections are more frequent and can be more chronic in primary malnourished individuals (2, 3), with evidence suggesting that infection further weakens the host by reducing nutrient uptake and impeding the ability to mount an effective immune response (4–6). However, relatively little is known about the physiological and immunological signatures linking general malnutrition or specific dietary deficiencies to infection susceptibility.

Vitamin B_12_ (cobalamin [Cbl]) serves as an essential cofactor in the cellular growth of most prokaryotic organisms. While bacterial species such as *Escherichia coli* and *Salmonella enterica* have the ability to produce Cbl *de novo* anaerobically, mammals obtain Cbl exclusively from animal protein dietary sources (7). Mammalian Cbl absorption is a highly specific process, with the secreted protein gastric intrinsic factor (Gif) being responsible for transporting Cbl through the small intestine and facilitating endocytosis in the distal ileum (8). In humans, Cbl deficiency is linked to a variety of clinical conditions, including megaloblastic anemia, optic atrophy, degeneration of the spinal cord, renal abnormalities, and malabsorption (8–11). The impact of Cbl deficiency is likely multifactorial, as it normally plays a role in cellular metabolism with DNA stress, cellular oxidative damage, alterations in odd-chain fatty acid and cholesterol synthesis, and anaplerosis as consequences (8, 12–15).

We have recently shown that mice lacking Gif exhibit significant growth retardation and low bone mass, with the penetrance of this phenotype showing maternal influences (16). Here we characterize the susceptibility of this mouse line to different infection challenges, highlighting maternal and metabolic influences on serum Cbl levels and susceptibility.

## RESULTS

### Gif-deficient mice were identified as part of a high-throughput screen as susceptible to a bacterial challenge.

**FIG 1:**
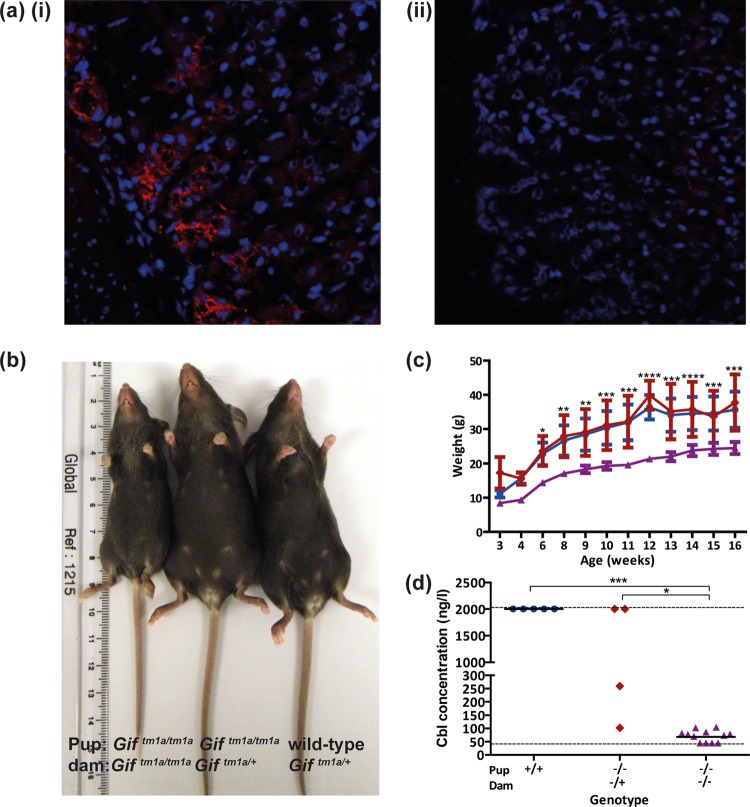
*Gif*^tm1a/tm1a^ mice exhibit signatures of Cbl deficiency. (a) Immunofluorescence analysis of the glandular stomach regions of wild-type (i) and *Gif*^tm1a/tm1a^ (ii) mice stained (red) for the presence of Gif with specific antiserum (×400 magnification). (b) Representative photograph of 8-week-old *Gif*^tm1a/tm1a^ and wild-type pups from *Gif*^tm1a/tm1a^ (left), *Gif*^tm1a/+^(middle), or wild-type (right) dams. (c) Mean body weights of F_1_ *Gif*^tm1a/tm1a^ (red triangles), F_2_ *Gif*^tm1a/tm1a^ (purple triangles), and wild-type (blue circles) mice between 3 and 16 weeks of age. (d) Blood plasma Cbl concentrations *Gif*^tm1a/tm1a^ and wild-type mice. Dashed lines show the detection limits of the analyzer. Black bars represent geometric mean values. ***, *P* < 0.001; **, *P* < 0.01; *, *P* < 0.05 (ANOVA with Dunn’s multiple-comparison *post hoc* test).

By high-throughput screening, we challenge small groups of novel reporter-tagged knockout mouse lines with different pathogens (17) (http://www.mousephenotype.org). One of the lines we screened harbored a defined mutation in the gene encoding Gif, a glycoprotein regulating the highly specific intestinal endocytosis of Cbl. *Gif*^tm1a/tm1a^ mice exhibit altered susceptibility to both *S. enterica* serovar Typhimurium and *Citrobacter rodentium* challenges (http://www.mousephenotype.org/data/genes/MGI:1202394). These *Gif* mutant mice were generated on a C57BL/6N background by using a knockout first promoter-driven allele targeting *Gif* intron 5 on chromosome 19 (see Text S1 in the supplemental material for further details). Transcriptional and immunohistological analyses showed that *Gif* expression was abolished in *Gif*^tm1a/tm1a^ mice and restricted to the stomachs of wild-type mice (16) (Fig. 1a shows the immunohistochemical analysis results obtained).

Subsequent breeding identified two phenotypically distinct types of *Gif*^tm1a/tm1a^ mice. F_1_ *Gif*^tm1a/tm1a^ mice resulting from *Gif*^tm1a/+^ × *Gif*^tm1a/+^ matings (Fig. 1b) were indistinguishable from heterozygous *Gif*^tm1a/+^ mice or their wild-type counterparts with respect to multiple characteristics, including their susceptibility to either an *S*. Typhimurium or a *C. rodentium* challenge (data not shown). In contrast, F_2_ *Gif*^tm1a/tm1a^ mice that resulted from *Gif*^tm1a/tm1a^ × *Gif*^tm1a/tm1a^ matings exhibited stunted growth (Fig. 1b and c) and enhanced susceptibility to both *S*. Typhimurium and *C. rodentium* challenges. We therefore examined these phenotypic characteristics further.

### F_2_ *Gif*^tm1a/tm1a^ mice are deficient in Cbl and have alterations in red blood cell and plasma chemistry.

To ascertain if *Gif*^tm1a/tm1a^ mice were Cbl deficient, we measured the blood plasma Cbl concentrations of wild-type and *Gif*^tm1a/tm1a^ mice (Fig. 1d). We found that all of the wild-type mice exhibited high plasma Cbl concentrations (≥2,000 ng/liter), with F_1_ *Gif*^tm1a/tm1a^ mice showing various levels of blood plasma Cbl deficiency. However, blood plasma from F_2_ *Gif*^tm1a/tm1a^ mice consistently exhibited at least 20-fold lower Cbl levels than that from their wild-type counterparts (Fig. 1d). Further blood analysis showed that naive F_2_ *Gif*^tm1a/tm1a^ mice exhibited phenotypes similar to those of humans with severe Cbl deficiency (9, 11), with these F_2_ mice having consistently lower erythrocyte counts (see Fig. S1b, part i, in the supplemental material) and a greater mean corpuscular volume (MCV; see Fig. S1b, part ii) than equivalent F_1_ *Gif*^tm1a/tm1a^ and wild-type mice. Moreover, megakaryocytes found in the spleens of F_2_ *Gif*^tm1a/tm1a^ mice were often hyperlobulated with hypersegmented nuclei indicative of cell cycle irregularities compared to wild-type cells (see Fig. S1a) (11).

Furthermore, in comparison to blood plasma collected from equivalent F_1_ *Gif*^tm1a/tm1a^ and wild-type mice, blood plasma from F_2_ *Gif*^tm1a/tm1a^ mice exhibited alterations in multiple clinical chemistry parameters (Fig. S1c). We observed that the concentrations of iron, high-density lipoproteins, cholesterol, as well as albumin, glucose, and glycerol, were significantly lower in the blood plasma of F_2_ *Gif*^tm1a/tm1a^ mice (Fig. S1c, parts i to vi). Interestingly the level of urea, a clinical marker of muscle damage, was found to be significantly higher in the blood plasma of F_2_ *Gif*^tm1a/tm1a^ mice than in that of F_1_ *Gif*^tm1a/tm1a^ and wild-type mice (see Fig. S2c, part vii, in the supplemental material).

### F_2_ *Gif*^tm1a/tm1a^ mice are hypersusceptible to *C. rodentium* and *S*. Typhimurium challenges.

**FIG 2:**
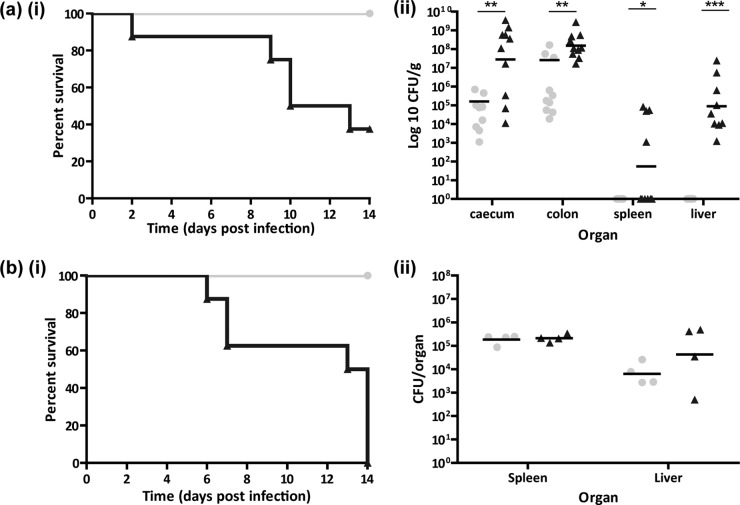
F_2_ *Gif*^tm1a/tm1a^ mice are susceptible to *C. rodentium* and *S*. Typhimurium pathogen challenges. (a, part i) Kaplan-Meier curve showing percent survival following infection of F_2_ *Gif*^tm1a/tm1a^ and wild-type mice with *C. rodentium* (*n* = 8). (a, part ii) Enumeration of *C. rodentium* bacteria in cecal, colon, spleen, and liver tissues of surviving F_2_ *Gif*^tm1a/tm1a^ and wild-type mice at day 14 p.i. (b, part i) Kaplan-Meier curve showing percent survival following infection of F_2_ *Gif*^tm1a/tm1a^ and wild-type mice with *S*. Typhimurium (*n* = 8). (b, part ii) Enumeration of *S*. Typhimurium bacteria in the spleens and livers of F_2_ *Gif*^tm1a/tm1a^ and wild-type mice at day 14 p.i. Gray circles represent wild-type mice. Black triangles represent F_2_ *Gif*^tm1a/tm1a^ mice. Black bars represent geometric mean values. ***, *P* < 0.001; **, *P* < 0.01; *, *P* < 0.05 (ANOVA with Dunn’s multiple-comparison *post hoc* test).

When groups of F_2_ *Gif*^tm1a/tm1a^ and wild-type mice were challenged with *C. rodentium*, 60% of the F_2_ *Gif*^tm1a/tm1a^ mice succumbed to infection by day 14 postinfection (p.i.), compared to none of the wild-type mice (Fig. 2a, part i). Although the enumeration of *C. rodentium* was statistically significantly higher in all of the organs of the challenged F_2_ *Gif*^tm1a/tm1a^ mice than in the challenged wild-type mice at day 14 p.i. (Fig. 2a, part ii), the two groups had similar colon weights and general patterns of colonic hyperplasia (see Fig. S2 in the supplemental material). Significantly, however, *C. rodentium* was consistently found (*P* < 0.001) in the livers of F_2_ *Gif*^tm1a/tm1a^ mice (Fig. 2a, part ii). Furthermore, other bacterial taxa, such as *Staphylococcus* and *Escherichia*, were also present in their livers, suggesting that polymicrobial sepsis was occurring after a *C. rodentium* challenge in these F_2_ *Gif*^tm1a/tm1a^ mice (data not shown). Importantly, bacteria were not generally detected in the livers of wild-type mice before or after a *C. rodentium* challenge.

Similar groups of wild-type and F_2_ *Gif*^tm1a/tm1a^ mice were also challenged with moderately attenuated *S*. Typhimurium M525. F_2_ *Gif*^tm1a/tm1a^ mice showed signs of salmonellosis by day 4 p.i., with all mice having to be sacrificed between days 6 and 14 p.i. (Fig. 2b, part i). In contrast, wild-type mice exhibited little sign of disease and survived the challenge. Interestingly, the *S*. Typhimurium burdens in the spleens and livers of F_2_ *Gif*^tm1a/tm1a^ and wild-type mice were comparable at day 14 p.i. (Fig. 2b, part ii). Thus, overwhelming *S*. Typhimurium burdens were unlikely to be responsible for the disease in F_2_ *Gif*^tm1a/tm1a^ mice.

### Histopathological signatures are observed in F_2_ *Gif*^tm1a/tm1a^ mice before and after a pathogen challenge.

**FIG 3:**
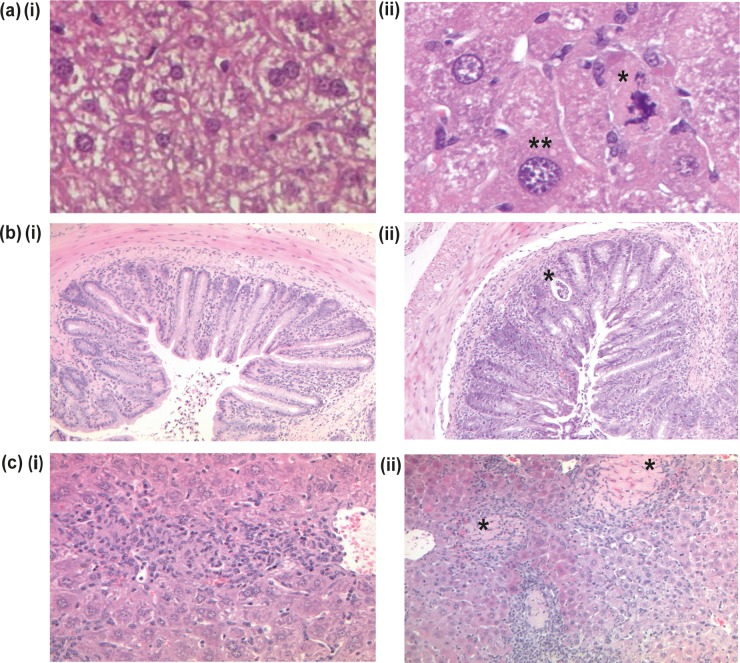
Representative hematoxylin- and eosin-stained sections from naive and infected F_2_ *Gif*^tm1a/tm1a^ and wild-type mice. (a) Liver sections from naive wild-type (i) and F_2_ *Gif*^tm1a/tm1a^ (ii) mice. A single asterisk indicates an abnormal mitotic figure, and double asterisks indicate enlarged cellular nuclei in an F_2_ *Gif*^tm1a/tm1a^ mouse (×400 magnification). (b) Colon sections obtained on day 14 after a *C. rodentium* challenge of wild-type (i) and F_2_ *Gif*^tm1a/tm1a^ (ii) mice. The asterisk indicates a crypt abscess in an F_2_ *Gif*^tm1a/tm1a^ mouse (×100 magnification). (c) Liver sections obtained on day 8 after an *S*. Typhimurium challenge of wild-type (i) and F_2_ *Gif*^tm1a/tm1a^ (ii) mice. Both images show inflammatory cellular infiltration and granuloma formation. Asterisks indicate the large necrotic regions seen in F_2_ *Gif*^tm1a/tm1a^ mice (× 200 magnification).

A detailed histopathological analysis of tissues from naive and pathogen challenged F_2_ *Gif*^tm1a/tm1a^ and wild-type mice was performed. The general colonic and splenic morphology of naive F_2_ *Gif*^tm1a/tm1a^ mice was predominantly indistinguishable from that of wild-type mice (see Fig. S3a and b in the supplemental material). In contrast, F_2_ *Gif*^tm1a/tm1a^ mouse livers had hepatocytes with enlarged nuclei and abnormal mitotic figures (Fig. 3a, part ii), indicating liver cell karyomegaly and mitotic impairment (18).

Although moderate inflammation and colonic pathology were present in wild-type mice at day 14 after a *C. rodentium* challenge (Fig. 3b, part i), F_2_ *Gif*^tm1a/tm1a^ mice developed a more severe epithelial inflammatory cell infiltration, accompanied by crypt abscesses containing neutrophils and intraepithelial lymphocytes in the mucosa and increased inflammation in the submucosa (Fig. 3b, part ii, for a higher magnification, and Fig. S3c, part i, in the supplemental material). Moreover, while the livers of wild-type mice challenged with *C. rodentium* showed no obviously enhanced inflammatory cell infiltrate, challenged F_2_ *Gif*^tm1a/tm1a^ mice exhibited rich inflammatory clusters particularly around the hepatic portal triad, with a thrombus occasionally present within the hepatic portal vein (see Fig. S3c, part ii).

The liver histology of F_2_ *Gif*^tm1a/tm1a^ mice challenged with *S*. Typhimurium exhibited evidence of significantly more damage than that of wild-type mice (Fig. 3c). Both groups of infected mice harbored clusters of macrophages forming granulomas; however, there were sizable areas of necrosis and regions of foamy fatty acid hepatocytes (fat vacuoles indicating metabolic stress) in the *S*. Typhimurium challenged F_2_ *Gif*^tm1a/tm1a^ mice (Fig. 3c, part ii). The spleens of wild-type *S*. Typhimurium infected mice displayed a normal distribution of red and white pulp, but similarly challenged F_2_ *Gif*^tm1a/tm1a^ mice exhibited increased extramedullary hematopoiesis with scattered large areas of necrosis with foci of macrophages in the red pulp compacting the white pulp (see Fig. S3d in the supplemental material).

### Immune profiling of naive and *S*. Typhimurium infected F_2_ *Gif*^tm1a/tm1a^ mice.

**FIG 4:**
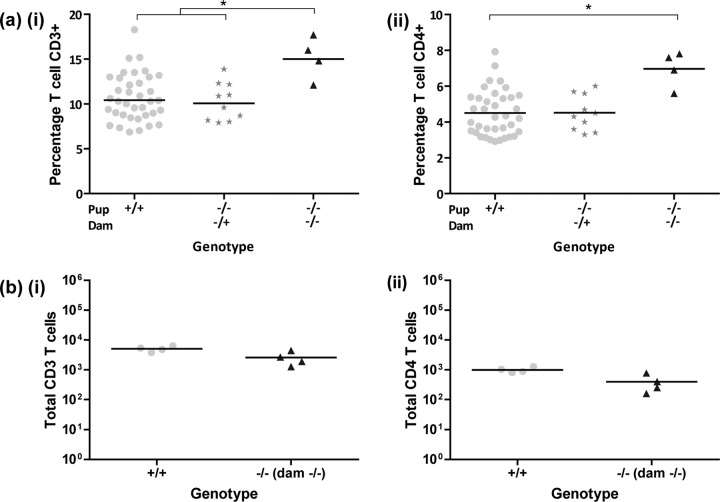
Immune cell profiling of naive and *S*. Typhimurium-challenged F_2_ *Gif*^tm1a/tm1a^ and wild-type mice. (a) Analysis of peripheral blood leukocytes from 16-week-old naive mice showing percentages of CD3^+^ (i) and CD4^+^ (ii) T cells. (b) Analysis of spleen cells from *S*. Typhimurium-infected mice on day 14 p.i. for total CD3^+^ (i) and total CD4^+^ (ii) T cells. Samples were analyzed on a BD LSR Fortessa or BD LSR II apparatus. Interpretation of the results was performed with FlowJo v9. Black bars represent geometric mean values. *, *P* < 0.05 (one-way ANOVA followed by Dunn’s multiple-comparison *post hoc* test).

We performed immune profiling before and after a challenge to identify immunological signatures associated with F_2_ *Gif*^tm1a/tm1a^ mouse susceptibility to *S*. Typhimurium (19). While we found that the percentages of peripheral blood phagocytes and natural killer cells were broadly comparable (http://www.mousephenotype.org/data/genes/MGI:1202394), we did observe slight differences in the percentages of peripheral blood CD4^+^ and CD8^+^ T cells and CD4^+^ CD25^+^ regulatory T cells (Fig. 4a, part ii, and Fig. S4a in the supplemental material) in F_2_ *Gif*^tm1a/tm1a^ mice and equivalent populations found in wild-type mice. However, when spleen leukocyte populations were analyzed at day 9 after *S*. Typhimurium infection, F_2_ *Gif*^tm1a/tm1a^ mouse T cell populations (Fig. 4b), as well as all of the other lymphocyte populations analyzed (see Fig. S4b), were comparable to those of similarly infected wild-type mice. Similarly, blood serum cytokine and chemokine analysis revealed no obvious differences between the concentrations of 25 different cytokines found in naive or infected F_2_ *Gif*^tm1a/tm1a^ mice and those in wild-type mice (see Text S1  in the supplemental material for further details).

As macrophages are critical for the control of *Salmonella* replication and the homeostasis of granulomas *in vivo* (20, 21), we isolated macrophages from the peritoneal cavities of naive F_2_ *Gif*^tm1a/tm1a^ and wild-type mice to evaluate their susceptibility to *S*. Typhimurium. We found that the ability of F_2_ *Gif*^tm1a/tm1a^ macrophages to kill *S*. Typhimurium was not impaired and was comparable to that of wild-type macrophages. Interestingly, we found that F_2_ *Gif*^tm1a/tm1a^ macrophages expressed higher levels of arginase 1 (see Fig. S4c in the supplemental material), a marker of alternatively activated M2 macrophages (6, 22).

### Transcriptional profiling indicates potential cellular, metabolic, and anaplerosis pathway abnormalities in F_2_ *Gif*^tm1a/tm1a^ mice.

To identify changes in gene expression that could have an impact on the phenotype of F_2_ *Gif*^tm1a/tm1a^ mice, microarray analysis was performed with mRNA prepared from the livers and colons of F_2_ *Gif*^tm1a/tm1a^ and wild-type mice with or without a pathogen challenge. Genes differentially expressed (*P* < 0.05 with a 0.8-log-fold difference between F_2_ *Gif*^tm1a/tm1a^ and wild-type mice) under each condition were further analyzed by Ingenuity Pathway Analysis (IPA; Qiagen, Redwood City, CA), enabling the clustering of dysregulated mRNAs to specific biological and canonical pathways. Further, IPA upstream regulator analysis was used to predict potential upstream transcriptional regulators likely controlling these biological function and canonical pathways in the transcriptional data of the F_2_ *Gif*^tm1a/tm1a^ mice.

Generally, differentially expressed genes in the colons of naive F_2_ *Gif*^tm1a/tm1a^ mice were related to lipid metabolism, cell-to-cell signaling, molecular transport, small molecule biochemistry, and cell death and survival processes (see Dataset S1b in the supplemental material). Upstream regulator analysis predicted that genes associated with glycolysis regulation (*Por*, *Clock*, *Cry1*, and *Cry2*) (23, 24) (see Dataset S1a) might be controlling the functional gene profile in the colons of naive F_2_ *Gif*^tm1a/tm1a^ mice. Functional gene categories in the livers of naive F_2_ *Gif*^tm1a/tm1a^ mice were largely associated with lipid, vitamin, mineral, and nucleic acid metabolism, as well as cellular molecular transport (see Dataset S1c), with upstream regulator analysis predicting glycolysis and fatty acid synthesis associated genes (*Gpd1*, *Slc25a13*, *RorC*, *RorA*, and *Acox1*; see Dataset S1a) as potential regulators of disrupted cellular and biological functions in the livers of naive F_2_ *Gif*^tm1a/tm1a^ mice. In particular, *Acox1* (encodes acyl coenzyme A [acyl-CoA] oxidase 1, palmitoyl), a peroxisome beta-oxidation fatty acid pathway regulator (25, 26), was predicted by IPA to be the most statically activated of the upstream transcriptional regulators (see Dataset S1a).

Of the differently expressed genes identified in colonic mRNA of F_2_ *Gif*^tm1a/tm1a^ mice challenged with *C. rodentium*, 60 were functionally associated with cell death and survival, with IPA predicting functional pathways associated with organismal injury and renal failure to be the most activated of these categories (see Dataset S1d in the supplemental material). Upstream transcriptional regulator analysis highlighted *Ptger4*, a prostaglandin regulator of mucosal integrity and suppressor of innate immunity (27), as comparatively activated (see Dataset S1a), with *Cfs2*, a modulator of epithelial cell homeostasis, as well as classical M1 macrophage activation (28, 29), as likely inhibiting canonical pathways in the colons of *C. rodentium*-infected F_2_ *Gif*^tm1a/tm1a^ mice (see Dataset S1a). The top five functional gene categories in hepatic mRNA of *C. rodentium*-infected F_2_ *Gif*^tm1a/tm1a^ mice were predicted to be related to cellular function and maintenance, movement, development, growth and proliferation, and signaling, with our analysis showing many of these associated genes relatively downregulated (see Dataset S1e). A key transcriptional regulator was predicted to be *Acox1* (see Dataset S1a).

Finally, IPA of the hepatic mRNA data of differentially expressed genes found in *S*. Typhimurium infected F_2_ *Gif*^tm1a/tm1a^ mice was performed (see Dataset S1a and f in the supplemental material). Consistent with the other IPA analyses (see Dataset S1a), *Acox1* was predicted to be a key regulatory element. Functional and canonical pathway analysis identified differentially expressed genes as associated with lipid, drug, and carbohydrate metabolism; small-molecule biochemistry; and cellular development. IPA indicated that functional pathways associated with fatty acid metabolism were overactivated (*P* = 4.39e^−06^; see Dataset S1e).

### Blood metabolic profiling identifies F_2_ *Gif*^tm1a/tm1a^ mice as having abnormal glycolytic pathways and cellular energy homeostasis pathways.

**FIG 5:**
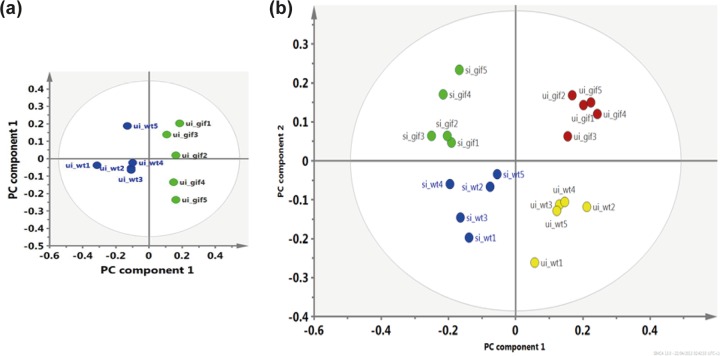
Metabotyping of blood serum from F_2_ *Gif*^tm1a/tm1a^ and wild-type mice reveals metabolic abnormalities. (a) PCA score plot separating naive F_2_ *Gif*^tm1a/tm1a^ mice (green circles) from naive wild-type mice (blue circles) with 92% of the variables explained (*R*^2^_cum_) by using the first four components with 72% predictability minus Q2. (b) Multivariate data analysis results comparing the blood serum of uninfected F_2_ *Gif*^tm1a/tm1a^ (ui_gif; red circles) and wild-type (ui_wt; yellow circles) mice and that of *S*. Typhimurium-infected F_2_ *Gif*^tm1a/tm1a^ (si_gif; green circles) and wild-type (si_wt; blue circles) mice. Principal component 1 discriminates on the basis of the presence or absence of infection, while principal component 2 separates different mouse genotypes, regardless of infection status. Ninety percent of the variables (*R*^2^_cum_) were explained by using the first five components with 73% predictability.

To identify metabolites that might be influenced downstream of the changes in transcriptional patterns observed in the tissues of F_2_ *Gif*^tm1a/tm1a^ mice, metabotyping was performed by nuclear magnetic resonance (NMR) spectroscopy of blood serum taken from F_2_ *Gif*^tm1a/tm1a^ and wild-type mice before and after an *S*. Typhimurium challenge. By performing principal component analysis (PCA), a multivariate data analysis method, we could distinguish the blood serum metabolic profiles of naive (Fig. 5a), as well as *S*. Typhimurium infected (Fig. 5b) F_2_ *Gif*^tm1a/tm1a^ and wild-type mice.

As expected, uninfected wild-type mice demonstrated blood metabolic patterns of normal cellular homeostasis, harboring higher levels of fatty acids and glucose (Table 1). In contrast, naive F_2_ *Gif*^tm1a/tm1a^ mice harbored a metabolic profile with similarities to the Warburg effect (30). This profile included higher levels of lactate and lower quantities of glucose, indicating that lactic acid fermentation was occurring to generate energy for cellular homeostasis. We also identified biomarkers of alternative cellular energy sourcing such a decrease amount of fatty acids and an increased quantity of citrate-aspartate, metabolites commonly associated with increased glycolysis and acetyl-CoA production (Table 1; see Fig. S5, in the supplemental material). Furthermore, we observed a high level of methylmalonate, a known plasma marker of Cbl deficiency, in the blood of naive F_2_ *Gif*^tm1a/tm1a^ mice, suggesting that Cbl deficiency could be disrupting the trichloroacetic acid (TCA) cycle (14, 15).

**TABLE 1:** Summary of the relative metabolic differences in the blood serum of naive and *S*. Typhimurium-infected F_2_ *Gif*^tm1a/tm1a^ and wild-type mice^a^

Metabolite(s)	Chemical shift(s) (ppm)	Fold change(s)^b^	
		Naive	Infected
Fatty acids	Several	↓−1.2	—, ↑1.4^c^
Putrescine-cadaverine	1.75, 3.04; 1.48, 3.80	—	↑
Glucose	3.24, 3.46, 3.52, 3.73; 3.83, 3.88, 5.22	↓−1.2	↓−1.3
Fucose	1.22, 1.25, 3.44, 3.60, 3.80, 5.25	—	↓−1.5
Acetate	1.93	↑	—
Alanine	1.49, 1.51, 3.81	↑	—
Taurine-betaine	3.27, 3.44; 3.25, 3.90	↑	↓−1.3, −2^c^
Citrate-aspartate	2.55, 2.67; 2.64, 2.70	↑1.2	—
Formate	8.47	↑	—
Glutamate-glutamine	(2.04, 2.12), (2.34, 2.39), 3.74; (2.00, 24.00, 2.48), 3.78	↓	—
Isoleucine	0.98, 1.01, 3.66	↓	—
Leucine	0.96, 1.72, 3.75	↓	↓−1.6
Methylmalonate	1.22, 3.18	↑1.3	↑1.2
Phenylacetylglycine	3.68, 3.76, 7.40	↑	↑
Valine	1.01, 1.06, 2.29, 3.61	—	↑
Adipate	1.56, 2.17	—	—, ↑1.6^c^
3-Hydroxybutyrate	1.21, (2.32, 2.42), 4.16	—	↓
Butyrate	0.89, 1.56, 2.15	—	↓
Lactate	1.34, 4.13	↑1.2	—
Pyruvate-succinate	2.39, 2.41	↑	—
Choline-phosphocholine- glycerophosphocholine	3.20, 3.21, 3.22	—	↑
Malate	(2.36, 2.42), (2.66, 2.70), 4.30	—	↑

a Fold change, >1.2; *P* < 0.05.

b Shown are fold (>1.2) and significant (*P* < 0.05) changes in metabolite levels in F_2_ *Gif*^tm1a/tm1a^ and wild-type mice.

c Shown are fold (>1.2) and significant (*P* < 0.05) changes in metabolite levels in naive versus *S*. Typhimurium-infected F_2_ *Gif*^tm1a/tm1a^ mice. Symbols: —, no significant fold change (>1.2); ↑  or ↓, relative increase or decrease in metabolites from PCA loading plots. These levels were estimated from relative intensities (median) of ^1^H NMR spectra following spectral normalization.

Next, we metabotyped blood serum from wild-type and F_2_ *Gif*^tm1a/tm1a^ mice at day 9 after an *S*. Typhimurium challenge (Fig. 5b). *S*. Typhimurium is known to cover its metabolic needs during infection by upregulating host cell glycolysis and associated fatty acid beta-oxidation pathways (22, 31, 32), with the blood of infected wild-type mice harboring relatively higher levels of metabolites indicative of beta oxidation activity such as 3-hydroxybutyrate and butyrate and adipate (Table 1). In contrast, challenged F_2_ *Gif*^tm1a/tm1a^ mice exhibited biomarkers associated with severe changes in cellular energy homeostasis and glycolytic processes, as well as metabolic starvation (30, 33). We identified lower levels of blood sugars (glucose and fucose), with taurine (Table 1), and leucine, metabolites thought to regulate maurine skeletal function as well as glucose and lipid homeostasis (12, 34) reduced in the blood of infected F_2_ *Gif*^tm1a/tm1a^ mice. We also observed biomarkers of increased acetyl-CoA and fatty acid synthesis (fatty acids, valine, and phenylacetylglycine), and increased oxidative stress (adipate, taurine-betaine, chlorine, and malate) (30). Furthermore, and comparative to their uninfected F_2_ *Gif*^tm1a/tm1a^ counterparts, the plasma Cbl marker methylmalonate was observed at high levels in the blood metabolite profile of infected F_2_ *Gif*^tm1a/tm1a^ mice (Table 1).

### Cbl supplementation reverses the *S*. Typhimurium susceptibility of F_2_ *Gif*^tm1a/tm1a^ mice.

**FIG 6:**
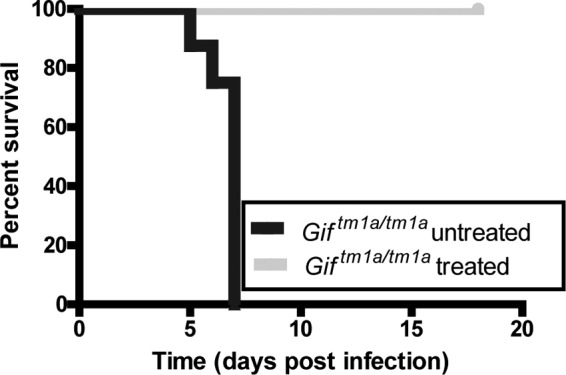
Cbl supplementation of F_2_ *Gif*^tm1a/tm1a^ mice alters their susceptibility to an *S*. Typhimurium challenge. Shown is a Kaplan-Meier curve of percent survival of F_2_ *Gif*^tm1a/tm1a^ mice left untreated or treated with cyanocobalamin and then infected with *S*. Typhimurium (*n* = 8).

To evaluate if F_2_ *Gif*^tm1a/tm1a^ Cbl deficiency was directly impacting their hypersusceptibility to *C. rodentium* and *S*. Typhimurium infection, we supplemented F_2_ *Gif*^tm1a/tm1a^ mice with Cbl in a series of subcutaneous injections prior to a challenge and compared their susceptibility to an *S*. Typhimurium M525 challenge with that of untreated F_2_ *Gif*^tm1a/tm1a^ mice. As expected, untreated F_2_ *Gif*^tm1a/tm1a^ mice showed signs of salmonellosis by day 4 p.i. and had to be sacrificed by day 7 p.i. (Fig. 6). In contrast, equivalent Cbl-treated F_2_ *Gif*^tm1a/tm1a^ mice exhibited limited signs of salmonellosis, survived the challenge, and were indistinguishable from their wild-type counterparts. Thus, Cbl deficiency in these F_2_ *Gif*^tm1a/tm1a^ mice is likely directly influencing their susceptibility to *S*. Typhimurium infection.

## DISCUSSION

By exploiting a novel *Gif* mutant mouse line, we have demonstrated a critical role for Cbl in controlling susceptibility to infection by two different bacterial pathogens, *C. rodentium* and *S*. Typhimurium. We link this severe phenotype to likely disruptions in glycolysis, fatty acid synthesis, and energy homeostasis processes in Cbl-defective mice (12, 14, 30, 31, 33), with infection susceptibility further being influenced by the genotype of mothers, a phenomenon linked to their ability to transfer Cbl to their offspring (35).

The results obtained with Gif deficient mice mimic aspects of the clinical phenotypes associated with Cbl deficiency in humans and other mammals. For example, children born to Cbl depleted mothers exhibit stunted growth (10), similar to that observed in F_2_ *Gif*^tm1a/tm1a^ mice and infant rats and mice when Cbl was removed from their diet (16, 35, 36). Further, we demonstrate how Cbl deficiency can likely disrupt mammalian cellular homeostasis processes. F_2_ *Gif*^tm1a/tm1a^ mice exhibited blood and histopathological signatures associated with DNA abnormalities such as megaloblastic anemia, abnormal mitotic figures, and cellular oxidative damage, phenotypes commonly associated with Cbl deficiency in humans and infant mice (8, 9, 11, 13). By metabolomic and transcriptional profiling, we also found evidence that succinyl-CoA, a Cbl by-product and an essential TCA cycle regulator, was inhibited in F_2_ *Gif*^tm1a/tm1a^ mice, likely leading to increased glycolysis and fatty acid synthesis and other metabolic activities commonly associated with the Warburg effect (12–15, 30).

We were unable to detect a clear immunological signature associated with Cbl deficiency in F_2_ *Gif*^tm1a/tm1a^ mice, even though others have reported that Cbl deficiency affects cytokine and T cell ratios in a uninfected host (37, 38). Clearly, we did not exhaustively screen for immune deficiencies, and further studies in this area are still warranted. However, the macrophage killing activity that is critical for controlling salmonellosis *in vivo* was not obviously impaired, and immune cell populations and cytokine levels critical for *Salmonella* control appeared to be broadly similar (19). Interestingly however, and perhaps consistent with the Warburg effect (6), we did identify signatures for alternatively activated M2 macrophages, such as increased expression of arginase 1 by F_2_ *Gif*^tm1a/tm1a^ mouse macrophages. We therefore concluded, like the authors of a previously published study (22), that this metabolic activation state has little impact on the control of *Salmonella* replication because these cells were still able to kill salmonellae *in vitro*.

After *C. rodentium* infection, F_2_ *Gif*^tm1a/tm1a^ mice exhibited polymicrobial sepsis, with an overgrowth of *C. rodentium*. Histopathological examination revealed greater recruitment of inflammatory cells to the colon and liver than similarly infected wild-type mice, with transcriptional profiling suggesting that cellular homeostasis processes are likely disrupted in F_2_ *Gif*^tm1a/tm1a^ mice after *C. rodentium* infection. F_2_ *Gif*^tm1a/tm1a^ mice infected with *S*. Typhimurium exhibited greater signs of lethargy and morbidity, with severe histopathological damage. As *S*. Typhimurium utilizes the same fatty acid beta-oxidation and glycolysis pathways that are already dysregulated in F_2_ *Gif*^tm1a/tm1a^ mice (22, 31, 32), we hypothesize that F_2_ *Gif*^tm1a/tm1a^ mice are succumbing to *S*. Typhimurium infection from metabolic starvation, with their metabolomic and transcriptional profile strongly resembling this scenario (33).

This study has further highlighted some of the complex clinical and metabolomic abnormalities associated with Cbl deficiency, demonstrating the importance of Cbl in the mammalian diet. By performing studies on *Gif*^tm1a/tm1a^ or other mice with genetic defects associated with nutrient absorption, we can not only gain a better biological understanding of the interplay between nutritional regulation and the immune system but also further understand the pathogenesis associated with specific nutritional dysregulation.

## MATERIALS AND METHODS

### Animals.

The *Gif*^tm1a(KOMP)Wtsi^ mouse line was generated on a C57BL/6N genetic background as part of the International Mouse Phenotyping Consortium (http://www.mousephenotyping.org). See Text S1  in the supplemental material for the gene targeting methods used to produce this mouse line. Animals were housed under specific-pathogen-free conditions in HEPA-filtered cages with sterile bedding and given a sterilized standard diet and water *ad libitum*. All experiments were performed in accordance with United Kingdom Animal (Scientific Procedures) Act 1986.

### Hematology and blood chemistry analysis.

Blood was collected under terminal anesthesia into either EDTA-coated tubes or heparinized tubes for plasma or serum preparation. Clinical chemistry analysis of plasma was performed with the Olympus AU400 analyzer (Beckman Coulter Ltd., High Wycombe, United Kingdom). Concentrations of Cbl in plasma were measured with the ADVIA Centaur immunoassay analyzer (Siemens).

### Cbl treatment.

Mice were treated subcutaneously with cyanocobalamin (Sigma Aldrich) at 1 mg/ml every 2 weeks for 6 weeks. Mice were left for 1 month before an *S*. Typhimurium infection challenge.

### Cytokine analysis.

Cytokine analysis was performed with the FlexMap 3D (Luminex) machine with a Milliplex Map Mouse Th17 Magnestic Bead Panel 96-well assay kit (Millipore). For method details, as well as a list of the cytokines analyzed, see Text S1 in the supplemental material.

### Flow cytometry analysis.

Analysis of peripheral blood leukocytes was performed with heparinized blood collected from 16-week-old mice. Peripheral blood leukocytes, mesenteric lymph nodes, and spleens were prepared and stained for flow cytometry analysis as described in Text S1 in the supplemental material. All samples were analyzed on an LSR II or LSR Fortessa apparatus (BD Biosciences). Data were analyzed with FlowJo v9 software (TreeStar).

### Histological analysis.

For histological examination, 5-µm sections of paraffin-embedded tissues were stained with hematoxylin and eosin (Sigma-Aldrich). Sections were examined and scored by a pathologist under blinded conditions. For immunohistochemistry analysis, sections were cut and fixed as described in Text S1 in the supplemental material. Sections were visualized by confocal microscopy (Leica).

### NMR analysis.

Blood serum was collected from age- and sex-matched mice (five per group), snap-frozen immediately, and stored at −80°C until analysis. Samples were analyzed with a Bruker ADVANCE III NMR spectrometer to identify metabolic differences between F_2_ *Gif*^tm1a/tm1a^ and wild-type mice. For further details, see Text S1 in the supplemental material.

### Microarray analysis.

mRNA for microarray analysis was prepared as described in Text S1 in the supplemental material. Analysis was performed with the Illumina MouseWG-6 v2.0 Expression BeadChip kit. The data were analyzed with BeadStudio Software (Illumina) to identify genes differently regulated in F_2_ *Gif*^tm1a/tm1a^ and wild-type mice. Further pathway analysis was performed by IPA (Qiagen, Redwood City, CA) of all of the genes differently expressed in F_2_ *Gif*^tm1a/tm1a^ and wild-type mice (*P* < 0.05; log-fold change, 0.8). For further details of the IPA analysis performed, see Text S1 in the supplemental material.

### RNA isolation and RT-qPCR of peritoneal macrophages.

Resident peritoneal macrophages were isolated from naive mice by peritoneal lavage. RNA was isolated with the RNeasy minikit (Qiagen) and reverse transcribed with the QuantiTect reverse transcription (RT) kit (Qiagen). RT-quantitative PCR (RT-qPCR) experiments were performed as described in Text S1 in the supplemental material.

### Mouse infection challenges.

Background matched wild-type and *gif*^tm1a(KOMP)Wtsi^ mice 6 to 10 weeks of age were maintained in accordance with United Kingdom Home Office regulations under project license PPL80/2099 and 80/2596 (2596 replaced 2099 upon expiry). The Wellcome Trust Sanger Institute Ethical Review Committee has also reviewed this license. For *C. rodentium* challenges, mice were infected orally with 0.2 ml of *C. rodentium* ICC180. For *S*. Typhimurium challenges, mice were infected intravenously with a sublethal dose of *S*. Typhimurium M525. All mice were monitored daily for defined humane endpoints in accordance with United Kingdom Home Office license guidelines.

### Measurement of pathogen burdens in organs.

Organs were aseptically removed and homogenized mechanically. Viable counts were determined by serially diluting the organ homogenates onto LB agar plates for CFU counting.

### Peritoneal macrophage gentamicin protection assay.

Peritoneal macrophages were infected with *S*. Typhimurium M525 at a multiplicity of infection (MOI) of 20 for 1 h and then treated with gentamicin for another hour. Cells were then washed and incubated in antibiotic-free Opti-MEM (Thermo Fisher) for a further 5 h. For quantitative analysis, cells were lysed in 1% Triton X-100, serially diluted, and plated onto LB plates for CFU counting.

### Statistical analysis.

Where not already stated in Text S1 in the supplemental material, the Student *t* test was performed for experiments comparing two groups. For data with more than two groups, statistical analysis was performed by a nonparametric one-way analysis of variance (ANOVA) with Dunn’s multiple-comparison *post hoc* test. For comparisons of groups with two or more factors, analysis was performed by two-way ANOVA with the Bonferroni multiple-comparison posttest. *P* < 0.05 was taken as significant in all cases. All tests were performed with GraphPad Prism 5 (GraphPad Software, Inc., La Jolla, CA).

### Microarray data accession numbers.

The microarray data associated with this paper are stored in the ArrayExpress public database (http://www.ebi.ac.uk/arrayexpress) under accession numbers E-MTAB-1879 and E-MTAB-1880.

## SUPPLEMENTAL MATERIAL

Text S1  Details of the materials and methods used in this study, including descriptions of the gene targeting and mouse production, Cbl analysis and treatment, hematology and blood chemistry analyses, flow cytometry analysis of immune cell populations, NMR analysis, microarray analysis, mouse infection challenges, measurement of pathogen burdens in organs, histological analysis, cytokine analysis, resident peritoneal macrophage assays, and statistical analyses. Download Text S1, DOCX file, 0.2 MB

Dataset S1  Summary of the mRNA transcriptional regulators and specific biological and conical pathways identified as part of IPA. This analysis was performed using microarray data comparing mRNA extracted from F_2_ *Gif*^tm1a/tm1a^ mice to mRNA extracted from wild-type mice (*P* < 0.05 with 0.8-log fold change). The mRNA was prepared from the livers and colons of F_2_ *Gif*^tm1a/tm1a^ and wild-type mice with or without a pathogen challenge. Download Dataset S1, XLS file, 0.4 MB

Figure S1  Histological, hematologic, and plasma defects are present in F_2_ *Gif*^tm1a/tm1a^ mice. (a) Representative hematoxylin and eosin stained spleen sections from naive F_2_ *gif*^tm1a/tm1a^ and wild-type mice showing that megakaryocytes were often hyperlobulated and hypersegmented in F_2_ *Gif*^tm1a/tm1a^ mice. Part i, wild-type mice; part ii, F_2_ *Gif*^tm1a/tm1a^ mice (×400 magnification). (b and c) Hematology and plasma chemistry analyses of F_2_ (black triangles), F_1_ (gray stars) *Gif*^tm1a/tm1a^, and wild-type (gray circles) mice at 16 weeks of age. (a, part i) Total red blood cell count per microliter, (ii) MCV of red blood cells in femtoliters. (b) Concentrations of iron (millimolar) (i), high density lipoproteins (millimolar) (ii), cholesterol (millimolar) (iii), glucose (nanomolar) (iv), glycerol (nanomolar) (v), albumin (grams per liter) (vi), and urea (nanomolar) (vi) in plasma. Black bars represent geometric mean values. ***, *P* < 0.001; **, *P* < 0.01; *, *P* < 0.05 (ANOVA with Dunn’s multiple-comparison *post hoc* test). Download Figure S1, DOCX file, 0.7 MB

Figure S2  (a) Colon mass (grams) and (b) average colonic crypt length (micrometers) of naive and *C. rodentium*-infected (day 14 p.i.) F_2_ *Gif*^tm1a/tm1a^ and wild-type mice. Gray circles represent wild-type mice, and black triangles represent F_2_ *Gif*^tm1a/tm1a^ mice. Black bars represent geometric mean values. Download Figure S2, DOCX file, 0.1 MB

Figure S3  Representative hematoxylin- and eosin-stained sections from naive and infected F_2_ *Gif*^tm1a/tm1a^ and wild-type mice. (a) Naive wild-type (i) and *Gif*^tm1a/tm1a^ (ii) mouse colon tissues (×100 magnification). (b) Naive wild-type (i) and F_2_ *Gif*^tm1a/tm1a^ (ii) mouse spleen tissues (×100 magnification). (c) F_2_ *Gif*^tm1a/tm1a^ mice 14 days after *C. rodentium* infection: i, colonic crypt abscess (*; ×400 magnification); ii, thrombi in the hepatic portal vein (**; ×200 magnification). This was not observed in the livers of wild-type mice (data not shown). (d) Spleen sections from wild-type mice (i) (×100 magnification) and F_2_ *Gif*^tm1a/tm1a^ mice (ii) on day 14 after *S*. Typhimurium infection. Asterisks indicate large necrotic areas not seen in wild-type infected mice (×200 magnification). Download Figure S3, DOCX file, 2.4 MB

Figure S4  Profiling of immune cells from naive F_1_ *Gif*^tm1a/tm1a^ (gray stars), F_2_ *Gif*^tm1a/tm1a^ (black triangles), and wild-type (gray circles) mice. (a) Peripheral blood lymphocyte analysis of percentages of regulatory T cells (i) and CD8^+^ cytotoxic T cells (ii). All samples were analyzed on a BD LSR II analyzer. (b) Analysis of wild-type and *Gif*^tm1a/tm1a^ mouse splenocytes on day 14 after *S*. Typhimurium infection. Shown are the total Gr-1 (i), F4/80 (ii), and B220 (iii) cells. All samples were analyzed on a BD LSR Fortessa analyzer. Interpretation of the results was performed with FlowJo (v9). (c) Resident peritoneal macrophages were isolated from naive wild-type and F_2_ *Gif*^tm1a/tm1a^ mice, and relative expression of the arginase 1-encoding gene was compared by RT-qPCR. (d) The microbicidal abilities of wild-type and F_2_ *Gif*^tm1a/tm1a^ mouse macrophages were compared by gentamicin protection assay. Thioglycolate-elicited peritoneal macrophages were infected with *S*. Typhimurium M525 at an MOI of 20 in serum-free medium, and 5 h after gentamicin treatment, cells were lysed and plated on agar plates in different dilutions and CFU were counted. Black bars represent geometric mean values. Statistical analysis was performed by ANOVA with Dunn’s multiple-comparison *post hoc* test for panels a and b and Student’s *t* test for panels c and d. *, *P* < 0.05; **, *P* < 0.01; ***, *P* < 0.001. Download Figure S4, DOCX file, 0.2 MB

Figure S5  High-resolution 600-MHz ^1^H NMR spectrum of blood serum from F_2_ *Gif*^tm1a/tm1a^ mice. Peaks: mobile fatty acids and lipoproteins [1, −CH_3_/cholesterol; 2, −(CH_2_)_n_−; 36, −CH_2_CH_2_CO; 11, −CH_2_C═C; 14, −CH_2_C═O; 20, ═C-(CH_2_)–C═; 25, -N(CH_3_)_3_]; 3, leucine and isoleucine; 4, valine; 5, 3-hydroxybutyrate; 6, methylmalonate; 7, lactate; 8, alanine; 9, cadaverine-putrescine; 10, acetate; 12, glutamine and glutamate; 13, methionine; 15, 3-hydroxybutyrate; 16, pyruvate; 17, succinate and malate; 18, glutamine; 19, citrate; 21, lysine-cadaverine; 22, creatine; 23, methylmalonate; 24, choline; 26, glucose; 27, glycine; 28, betaine-taurine; 29, serine; 30, phenylacetylglycine; 31, alpha-glucose; 32, mobile unsaturated lipids; 33, fumarate; 34, tyrosine; 35, phenylalanine; 37, allantoin. Download Figure S5, DOCX file, 0.3 MB

## References

[1] Okoro CK, Kingsley RA, Quail MA, Kankwatira AM, Feasey NA, Parkhill J, Dougan G, Gordon MA 2012 High-resolution single nucleotide polymorphism analysis distinguishes recrudescence and reinfection in recurrent invasive nontyphoidal Salmonella typhimurium disease. Clin Infect Dis 54:955–963. doi:10.1093/cid/cir1032. 22318974 PMC3297646

[2] Mondal D, Minak J, Alam M, Liu Y, Dai J, Korpe P, Liu L, Haque R, Petri WA Jr. 2012 Contribution of enteric infection, altered intestinal barrier function, and maternal malnutrition to infant malnutrition in Bangladesh. Clin Infect Dis 54:185–192. doi:10.1093/cid/cir807. 22109945 PMC3245731

[3] Cunningham-Rundles S, McNeeley DF, Moon A 2005 Mechanisms of nutrient modulation of the immune response. J Allergy Clin Immunol 115:1119–1128; quiz 1129. doi:10.1016/j.jaci.2005.04.036. 15940121

[4] Maggini S, Wintergerst ES, Beveridge S, Hornig DH 2007 Selected vitamins and trace elements support immune function by strengthening epithelial barriers and cellular and humoral immune responses. Br J Nutr 98(Suppl 1):S29–S35. doi:10.1017/S0007114507832971. 17922955

[5] Rodríguez L, Cervantes E, Ortiz R 2011 Malnutrition and gastrointestinal and respiratory infections in children: a public health problem. Int J Environ Res Public Health 8:1174–1205. doi:10.3390/ijerph8041174. 21695035 PMC3118884

[6] Kelly B, O’Neill LA 2015 Metabolic reprogramming in macrophages and dendritic cells in innate immunity. Cell Res 25:771–784. doi:10.1038/cr.2015.68. 26045163 PMC4493277

[7] Martens JH, Barg H, Warren MJ, Jahn D 2002 Microbial production of vitamin B_12_. Appl Microbiol Biotechnol 58:275–285. doi:10.1007/s00253-001-0902-7. 11935176

[8] Quadros EV 2010 Advances in the understanding of cobalamin assimilation and metabolism. Br J Haematol 148:195–204. doi:10.1111/j.1365-2141.2009.07937.x. 19832808 PMC2809139

[9] O’Leary F, Samman S 2010 Vitamin B_12_ in health and disease. Nutrients 2:299–316. doi:10.3390/nu2030299. 22254022 PMC3257642

[10] Muthayya S, Kurpad AV, Duggan CP, Bosch RJ, Dwarkanath P, Mhaskar A, Mhaskar R, Thomas A, Vaz M, Bhat S, Fawzi WW 2006 Low maternal vitamin B_12_ status is associated with intrauterine growth retardation in urban south Indians. Eur J Clin Nutr 60:791–801. doi:10.1038/sj.ejcn.1602383. 16404414

[11] Aslinia F, Mazza JJ, Yale SH 2006 Megaloblastic anemia and other causes of macrocytosis. Clin Med Res 4:236–241. doi:10.3121/cmr.4.3.236. 16988104 PMC1570488

[12] Green CR, Wallace M, Divakaruni AS, Phillips SA, Murphy AN, Ciaraldi TP, Metallo CM 2016 Branched-chain amino acid catabolism fuels adipocyte differentiation and lipogenesis. Nat Chem Biol 12:15–21. doi:10.1038/nchembio.1961. 26571352 PMC4684771

[13] Ahmad S, Kumar KA, Basak T, Bhardwaj G, Yadav DK, Lalitha A, Chandak GR, Raghunath M, Sengupta S 2013 PPAR signaling pathway is a key modulator of liver proteome in pups born to vitamin B(12) deficient rats. J Proteomics 91:297–308. doi:10.1016/j.jprot.2013.07.027. 23928364

[14] Frenkel EP, Kitchens RL, Johnston JM 1973 The effect of vitamin B_12_ deprivation on the enzymes of fatty acid synthesis. J Biol Chem 248:7450–7456. 4147681

[15] Tanpaiboon P 2005 Methylmalonic acidemia (MMA). Mol Genet Metab 85:2–6. 15959932 10.1016/j.ymgme.2005.03.008

[16] Roman-Garcia P, Quiros-Gonzalez I, Mottram L, Lieben L, Sharan K, Wangwiwatsin A, Tubio J, Lewis K, Wilkinson D, Santhanam B, Sarper N, Clare S, Vassiliou GS, Velagapudi VR, Dougan G, Yadav VK 2014 Vitamin B_12_-dependent taurine synthesis regulates growth and bone mass. J Clin Invest 124:2988–3002. doi:10.1172/JCI72606. 24911144 PMC4071367

[17] White JK, Gerdin AK, Karp NA, Ryder E, Buljan M, Bussell JN, Salisbury J, Clare S, Ingham NJ, Podrini C, Houghton R, Estabel J, Bottomley JR, Melvin DG, Sunter D, Adams NC, Sanger Institute Mouse Genetics Project, Tannahill D, Logan DW, Macarthur DG, Flint J, Mahajan VB, Tsang SH, Smyth I, Watt FM, Skarnes WC, Dougan G, Adams DJ, Ramirez-Solis R, Bradley A, Steel KP 2013 Genome-wide generation and systematic phenotyping of knockout mice reveals new roles for many genes. Cell 154:452–464. doi:10.1016/j.cell.2013.06.022. 23870131 PMC3717207

[18] Schatten H, Wiedemeier AM, Taylor M, Lubahn DB, Greenberg NM, Besch-Williford C, Rosenfeld CS, Day JK, Ripple M 2000 Centrosome-centriole abnormalities are markers for abnormal cell divisions and cancer in the transgenic adenocarcinoma mouse prostate (TRAMP) model. Biol Cell 92:331–340. doi:10.1016/S0248-4900(00)01079-0. 11071042

[19] Dougan G, John V, Palmer S, Mastroeni P 2011 Immunity to salmonellosis. Immunol Rev 240:196–210. doi:10.1111/j.1600-065X.2010.00999.x. 21349095

[20] Monack DM, Raupach B, Hromockyj AE, Falkow S 1996 Salmonella typhimurium invasion induces apoptosis in infected macrophages. Proc Natl Acad Sci U S A 93:9833–9838. doi:10.1073/pnas.93.18.9833. 8790417 PMC38515

[21] Sheppard M, Webb C, Heath F, Mallows V, Emilianus R, Maskell D, Mastroeni P 2003 Dynamics of bacterial growth and distribution within the liver during salmonella infection. Cell Microbiol 5:593–600. doi:10.1046/j.1462-5822.2003.00296.x. 12925129

[22] Eisele NA, Ruby T, Jacobson A, Manzanillo PS, Cox JS, Lam L, Mukundan L, Chawla A, Monack DM 2013 Salmonella require the fatty acid regulator PPARdelta for the establishment of a metabolic environment essential for long-term persistence. Cell Host Microbe 14:171–182. doi:10.1016/j.chom.2013.07.010. 23954156 PMC3785333

[23] Otto DM, Henderson CJ, Carrie D, Davey M, Gundersen TE, Blomhoff R, Adams RH, Tickle C, Wolf CR 2003 Identification of novel roles of the cytochrome P450 system in early embryogenesis: effects on vasculogenesis and retinoic acid homeostasis. Mol Cell Biol 23:6103–6116. doi:10.1128/MCB.23.17.6103-6116.2003. 12917333 PMC180925

[24] Li MD, Li CM, Wang Z 2012 The role of circadian clocks in metabolic disease. Yale J Biol Med 85:387–401. 23012586 PMC3447202

[25] Fournier B, Saudubray JM, Benichou B, Lyonnet S, Munnich A, Clevers H, Poll-The BT 1994 Large deletion of the peroxisomal acyl-CoA oxidase gene in pseudoneonatal adrenoleukodystrophy. J Clin Invest 94:526–531. doi:10.1172/JCI117365. 8040306 PMC296126

[26] Li Y, Tharappel JC, Cooper S, Glenn M, Glauert HP, Spear BT 2000 Expression of the hydrogen peroxide-generating enzyme fatty acyl CoA oxidase activates NF-kappaB. DNA Cell Biol 19:113–120. doi:10.1089/104454900314627. 10701777

[27] Kabashima K, Saji T, Murata T, Nagamachi M, Matsuoka T, Segi E, Tsuboi K, Sugimoto Y, Kobayashi T, Miyachi Y, Ichikawa A, Narumiya S 2002 The prostaglandin receptor EP4 suppresses colitis, mucosal damage and CD4 cell activation in the gut. J Clin Invest 109:883–893. doi:10.1172/JCI14459. 11927615 PMC150928

[28] Enzler T, Gillessen S, Manis JP, Ferguson D, Fleming J, Alt FW, Mihm M, Dranoff G 2003 Deficiencies of GM-CSF and interferon gamma link inflammation and cancer. J Exp Med 197:1213–1219. doi:10.1084/jem.20021258. 12732663 PMC2193978

[29] Dongari-Bagtzoglou A, Kashleva H 2003 Granulocyte-macrophage colony-stimulating factor responses of oral epithelial cells to Candida albicans. Oral Microbiol Immunol 18:165–170. doi:10.1034/j.1399-302X.2003.00061.x. 12753468

[30] Vander Heiden MG, Cantley LC, Thompson CB 2009 Understanding the Warburg effect: the metabolic requirements of cell proliferation. Science 324:1029–1033. doi:10.1126/science.1160809. 19460998 PMC2849637

[31] Arsenault RJ, Napper S, Kogut MH 2013 Salmonella enterica Typhimurium infection causes metabolic changes in chicken muscle involving AMPK, fatty acid and insulin/mTOR signaling. Vet Res 44:35. doi:10.1186/1297-9716-44-35. 23682635 PMC3663815

[32] Antunes LC, Arena ET, Menendez A, Han J, Ferreira RB, Buckner MM, Lolić P, Madilao LL, Bohlmann J, Borchers CH, Finlay BB 2011 Impact of salmonella infection on host hormone metabolism revealed by metabolomics. Infect Immun 79:1759–1769. doi:10.1128/IAI.01373-10. 21321075 PMC3067560

[33] Lempradl A, Pospisilik JA, Penninger JM 2015 Exploring the emerging complexity in transcriptional regulation of energy homeostasis. Nat Rev Genet 16:665–681. doi:10.1038/nrg3941. 26460345

[34] De Luca A, Pierno S, Camerino DC 2015 Taurine: the appeal of a safe amino acid for skeletal muscle disorders. J Transl Med 13:243. doi:10.1186/s12967-015-0610-1. 26208967 PMC4513970

[35] Hellegers A, Okuda K, Nesbitt RE Jr., Smith DW, Chow BF 1957 Vitamin B_12_ absorption in pregnancy and in the newborn. Am J Clin Nutr 5:327–331. 13424485 10.1093/ajcn/5.3.327

[36] Molina V, Medici M, Taranto MP, Font de Valdez G 2008 Effects of maternal vitamin B_12_ deficiency from end of gestation to weaning on the growth and haematological and immunological parameters in mouse dams and offspring. Arch Anim Nutr 62:162–168. doi:10.1080/17450390801892567. 18459540

[37] Funada U, Wada M, Kawata T, Mori K, Tamai H, Isshiki T, Onoda J, Tanaka N, Tadokoro T, Maekawa A 2001 Vitamin B-12-deficiency affects immunoglobulin production and cytokine levels in mice. Int J Vitam Nutr Res 71:60–65. doi:10.1024/0300-9831.71.1.60. 11276924

[38] Tamura J, Kubota K, Murakami H, Sawamura M, Matsushima T, Tamura T, Saitoh T, Kurabayshi H, Naruse T 1999 Immunomodulation by vitamin B_12_: augmentation of CD8^+^ T lymphocytes and natural killer (NK) cell activity in vitamin B_12_-deficient patients by methyl-B_12_ treatment. Clin Exp Immunol 116:28–32. doi:10.1046/j.1365-2249.1999.00870.x. 10209501 PMC1905232

