# Supplementary material for: Infection Susceptibility in Gastric Intrinsic Factor (Vitamin B12)-Defective Mice Is Subject to Maternal Influences
Source: mBio. 2016 Jun 21;7(3):e00830-16. doi: 10.1128/mBio.00830-16 (PMC4916386; doi:10.1128/mBio.00830-16)
Supplement: Text S1 — Details of the materials and methods used in this study, including descriptions of the gene targeting and mouse production, Cbl analysis and treatment, hematology and blood chemistry analyses, flow cytometry analysis of immune cell populations, NMR analysis, microarray analysis, mouse infection challenges, measurement of pathogen burdens in organs, histological analysis, cytokine analysis, resident peritoneal macrophage assays, and statistical analyses. Download [file mbo003162863s1.docx]

**SUPPLEMENTAL MATERIAL for manuscript:**

Infection susceptibility in gastric intrinsic factor (Vitamin B_12_) defective mice is subject to maternal influences.

Lynda Mottram,^a^ Anneliese O. Speak,^a^ Reza M. Selek,^b,c^ Emma L. Cambridge,^a^ Zoe McIntyre,^a^ Leanne Kane,^a^ Subhankar Mukhopadhyay,^a^ Carolyn Grove,^a^ Amy Colin,^a^ Cordelia Brandt,^a^ Maria A. Duque-Correa,^a^ Jessica Forbester,^a^ Tu Anh Pham Nguyen,^a^ Christine Hale,^a^ George S. Vasilliou,^a^ Mark J. Arends,^d^ Brendan W. Wren,^e^ Gordon Dougan,^a^  Simon Clare^a^

Wellcome Trust Sanger Institute, Wellcome Trust Genome Campus, Hinxton, UK^a^; Wellcome Trust Genome Campus, European Bioinformatics Institute, Cheminformatics and Metabolism, Hinxton, UK^b^; Department of Biochemistry and the Cambridge Systems Biology Centre, University of Cambridge, Cambridge, UK^c^; Department of Pathology, University of Edinburgh, Edinburgh, UK ^d^; Department of Pathogen Molecular Biology, London School of Hygiene and Tropical Medicine, London, UK^e^.

Address correspondence to Simon Clare, [SC7@sanger.ac.uk](mailto:SC7@sanger.ac.uk).

**SUPPLEMENTAL METHODS**

**Gene targeting and mouse production.** Gene targeting was performed as part of the International Knockout Mouse Consortium ([www.knockoutmouse.org](http://www.knockoutmouse.org)) using high throughput methods (1, 2). The *Gif* mouse line was derived from the EPD0099_4_B07 ES cell clone and maintained on a C57BL/6N genetic background. The care and the use of the mice were in accordance with the United Kingdom Animal (Scientific Procedures) Act, 1986. The mice were maintained in specific pathogen free conditions and were housed in high efficiency particulate air filtered cages, with sterile bedding and given sterilized standard diet and water *ad libitum*. For genotyping, DNA was extracted using the DNeasy Blood and Tissue Kit (Qiagen), and PCR was performed using Platinum PCR Supermix (Invitrogen) and the following primers from Sigma-Aldrich:

*GIF*_F: TCTTCTCGGGGATCAAGAGC,

*GIF*_R: GTCACCTTGGTCTTCCCAGC,

Cassette_R: TCGTGGTATCGTTATGCGCC,

β-gal_F: ATCACGACGCGCTGTATC

β-gal_R: ACATCGGGCAAATAATATCG.

Three reactions were performed to confirm the genotype: (i) for GIF wild-type allele with primers GIF**_**F and GIF_R, (ii) for GIF targeted allele with primers GIF_F and Cassette_R, and (iii) for the β-galactosidase reporter cassette using the β-gal_F and β-gal_R primer pair (1).

**Cobalamin analysis and treatment.** Blood was collected via cardiac heart puncture and plasma was prepared and analysed on the ADVIA Centaur Immunoassay analyser (Siemens) by the Clinical Biochemistry and Immunology team at Addenbrooke’s Hospital, Cambridge, UK to determine concentration of Cbl in the plasma (ng/l).

Mice were treated subcutaneously with 1mg/ml of cyanocobalamin (Sigma Aldrich) every two weeks for a period of 6 weeks. The mice were then left for one month before *S*. Typhimurium infection challenge.

**Haematology and blood chemistry.** At 16 weeks of age, blood was collected from mice by puncture of the retro-orbital sinus under terminal anaesthesia into EDTA-coated tubes (Kabe Labortechnik GmbH, Numbrecht, Germany) for haematology (Scil Vetabc, Montpellier, France) and into heparinised tubes (Kabe Labortechnik GmbH) for plasma preparation. Plasma was analysed using an Olympus AU400 analyser (Beckman Coulter Ltd, High Wycombe, UK) with reagents from Beckman Coulter, Wako and Roche.

**Flow cytometry analysis of immune cell populations.** Flow cytometric analysis of peripheral blood leukocytes was performed on heparinised blood collected from 16-week old mice. After removal of plasma the buffy coat was removed and red blood cells were lysed by addition of BD Pharmlyse solution followed by washing with FACS buffer (PBS with 0.5% BSA). After blocking of Fc receptors with BD FcBlock for 10 min on ice fluorescently conjugated titrated antibodies were added and incubated for 20 min on ice. The following antibodies were used anti-CD4 (RM4-5, BD Pharmingen), anti-CD3 (17A2, eBioscience), anti-CD25 (PC61, BD Pharmingen), anti-CD8α (53-6-7, BD Pharmingen), anti-NK1.1 (PK136, BD Pharmingen), anti-CD44 (IM7, BD Pharmingen), anti-CD62L (MEL-14, Abcam), anti-CD19 (ID3, BD Pharmingen), anti-CD11b (M1/70, Catlag), anti-Gr1 (RB6-8C5, BD Pharmingen), anti-IgD (11-26c.2a, BD Pharmingen) and anti-CD45 (30F11, Biolegend). After staining propidium iodide (Sigma) solution was added to a final concentration of 2.5 mg/ml and incubated for a further 5 min on ice. After washing three times with FACS buffer cells were resuspended in FACS buffer and analysed on a BD LSR II. Dead cells were excluded on the basis of propidium iodide staining and relative frequencies of total T cells, CD4+ and CD8+ T cells, NKT cells, NK cells, B cells, granulocytes and monocytes in the total CD45+ WBC population were determined after analysis in FlowJo software (v9.5, TreeStar Inc, Ashland, OR, USA). Percentages of memory and regulatory CD4 T cells are presented relative to the total CD4+ T cell population. Percentages of memory CD8 are presented relative to the total CD8+ T cells and mature IgD+ B cells are relative to total B cell population. Mesenteric lymph node or spleen were prepared for analysis as previously described (3, 4). All samples were analysed on an LSR II or LSR Fortessa (BD Biosciences). Data was analysed using Flowjo v9 software (TreeStar).

**NMR analysis.** Blood serum was collected from age and sex matched mice (five mice per group), snap frozen immediately and stored at -80°C until analysis. Upon analysis, samples were prepared in isotonic saline containing D_2_O, pH 7.4, and run accordingly on NMR. Spectra have been previously acquired on a 11.7 Tesla/500 MHz superconducting magnet interfaced with a Bruker ADVANCE III NMR spectrometer using a 5 mm TXI ATMA inverse probe (BrukerBioSpin GmbH, Rheinstetten, Germany). All 1-dimensional (1D) spectra were acquired at 310 Kelvin with a spectral width of 16.00 ppm using a conventional pre-saturation pulse sequence for water suppression based on the first increment of the Nuclear Overhauser Effect Spectroscopy (NOESY) pulse sequence [RD (relaxation delay) – p/2 – t1 –p/2 – t_m_ – p/2 acquire where t_m_ = mixing time)]. The water resonance was irradiated during the relaxation delay (2.0 s) and mixing time (t_m_=50 ms), with t1 fixed at 4 ms. Each spectrum was acquired with 196 scans collected into 64 k data points with an acquisition time of 4.089 s. All spectra were processed using 1D NMR Manager software (version 12, Advanced Chemistry Development Inc., Toronto, Canada), zero-filled to twice the number of points and multiplied by an exponential weighting function corresponding to a line broadening of 0.3 Hz, Fourier transformed, phased, baseline corrected and referenced to alpha-glucose anomeric doublet at 5.25. Spectra were segmented into variable length bucketing (frequency windows) averaging 0.03 ppm chemical shift between 0.2 and 4.40 ppm (excluding water resonance) and 5.15 to 8.85 ppm using the Intelligent Bucketing facility (a variable length bucket integration system designed to ensure peaks do not straddle buckets). To account for differences in sample amounts each integrated region was normalised to the total spectral area. Spectra were assigned by comparison with previous literature (5-7) using Chenomix NMR Suite version 7.5 (Chenomix, Inc. Edmonton, Alberta, Canada) database with peak fitting capabilities. Multivariate pattern recognition techniques were used to analyse the NMR data set, since this method is capable of handling multiple variables simultaneously and to cope with numerous co-linearities and missing variables unlike univariate approaches. This was carried out using SIMCA-P+ 13.0 (Umetrics AB, Umeå, Sweden). Data were mean centred and Pareto scaled prior to analysis. Several multivariate statistical techniques were used within the SIMCA package; principal components analysis (PCA) and projection to latent structures by partial least squares discriminant analysis (PLS-DA (data not shown)) and Hierarchical Cluster Analysis (HCA) (8). The quality of the models were described by *R^2^* and *Q^2^* values with *R^2^* defined as the proportion of variances in the data explained by the models and goodness of fit, and *Q^2^* defined as the proportion of data variances predictable by the model and indicated predictability. Statistical significance was evaluated for individual values using t test for unequal variances.

### Microarray analysis. mRNA samples were quantified using NanoDrop 1000 (ThermoScientific), and quality-checked by analysis on an Agilent 2100 Bioanalyzer (Agilent Technologies). 500ng of RNA from each samplewas amplified using the Illumina TotalPrep RNA Amplification Kit (Ambion). The biotinylated cRNA in hybridization buffer was then applied to Illumina Mouse WG-6 v2 Expression BeadChips, and processed according to standard Illumina protocols. The slides were scanned using an Illumina BeadArray Reader. The data was analysed using BeadStudio Software (Illumina) to identify differently regulated genes in the F2 *Gif ^tm1a/tm1a^* mice compares to wild-type mice. Upstream gene regulator analysis was performed using Qiagen’s Ingenuity^®^ Pathway Analysis Upstream Regulator analytic tool (IPA^®^, Qiagen Redwood city, www.qiagen.com/ingenuity) on all differently expressed genes in the F2 *Gif ^tm1a/tm1a^* mice compared to wild-type mice (P<0.05, log fold change 0.8) that were identified using BeadStudio Software analysis. The functional and canonical analysis of IPA identified the significant biological functions and pathways with P<0.05 (Fisher’s exact test) considered significant. The upstream regulator analysis function of IPA was used to identify potential transcriptional regulators that could explain the observed changes in gene expression in F2 *Gif ^tm1a/tm1a^* mice. The IPA activation Z score was calculated on predicted activation or inhibition of transcriptional regulators based on published findings accessible through IPA Ingenutiy^®^ knowledge data base. Regulators or pathways with z-scores greater than 2 or less than -2 were considered to be significantly activated/increased or inhibited/decreased by IPA analysis. The microarray datasets associated with this paper are stored in the array express public database (www.ebi.ac.uk/arrayexpress) under the accession numbers E-MTAB-1879 and E-MTAB-1880.

## **Mouse infection challenges** Background-matched wild-type and *Gif ^tm1a/tm1a^* mice 6–10 weeks of age were maintained in accordance with UK Home Office regulations under the project licence PPL80/2099 and 80/2596 (2596 replaced 2099 upon expiry). This licence was reviewed by The Wellcome Trust Sanger Institute Ethical Review Committee. For the *C. rodentium* challenge, mice were infected orally with 0.2ml *C. rodentium* ICC180. For *S*. Typhimurium challenge, mice were infected intravenously with a sub-lethal dose of *S.* Typhimurium M525. All mice were monitored daily using the defined humane end points in accordance with UK Home Office licence guidelines.

Measurement of pathogen burdens in organs. Organs were aseptically removed and homogenized mechanically. Viable counts of organ homogenates were determined by serially diluting homogenates on to LB agar plates containing naladixic acid and kanamycin (100μg/ml) for *C. rodentium* lux ICC180 infected mice or LB agar plates containing ampicillin (100μg/ml) for *S*. Typhimurium M525 infected mice. Plates were incubated at 37˚C overnight and bacterial colonies enumerated.

**Histological analysis.** For histological examination of tissues, 5μm sections of paraffin-embedded tissue were stained with haematoxylin and eosin (Sigma-Aldrich). Sections were examined and scored by a pathologist under blinded conditions. For immunohistochemistry, 5μm sections were cut and air fixed onto glass slides before being fixed in 100% acetone. Sections were treated with blocking buffer (10% goat serum, 5% fish gelatine, 0.01% sodium azide, 0.1% BSA, and 0.01% Tween-20) for one hour. The blocking buffer was tapped off, and washed three times in PBS. An anti-intrinsic factor (Gif) antibody (Abcam) was added at a concentration of 1:50 in blocking buffer. Slides were incubated at room temperature for one hour in darkness, and then washed three times with PBS. The sections were then incubated with a Cy3 antibody (Invitrogen) at a concentration of 1:2000 in blocking buffer and re-incubated at room temperature for one hour in darkness. Slides were washed three times in PBS before being mounted using ProLong Gold Antifade Reagent (Invitrogen), covered with a glass slip and left to air dry in darkness at 4˚C overnight. Coverslips were fixed to the slides using nail varnish and left to dry. Sections were visualised using a Leica confocal microscope.

**Cytokine analysis.** Blood was left to clot for ten minutes and then serum was prepared by centrifugation. 0.5cm^2^ of colon from the apitipical tip, were dissected from each mouse and cut open longitudinately to reveal the lumen. Samples were washed in PBS, and RPMI^+^ medium (RPMI media supplemented with ten per cent foetal calf serum, two millimolar of glutamine, 100 µl/ml of penicillin, and 100 μg/ml of streptomycin). Samples were incubated at 37˚C for 12 hours. The RPMI^+^ medium was extracted for analysis. Cytokines were analysed on the FlexMap 3D (Luminex) machine using a Milliplex Map Mouse Th17 Magnestic Bead Panel 96 well assay kit (Millipore) as per manufacturer’s instruction. The cytokines in the Th17 panel were; CD40 Ligand, TNFβ, TNF-α, MIP-3α/CCL20, IL-33, IL-31, IL-28B, IL-27, IL-23, IL-22, IL-21, IL-17F, IL-17E/IL-25, IL-17A, IL-15, IL-13, IL-12 (p70), IL-10, IL-6, IL-5, IL-4, IL-2, IL-1β, IFN-γ, GM-CSF.

**RNA isolation and RT-qPCR of peritoneal macrophages.** Resident peritoneal macrophages from naive mice were isolated by peritoneal lavage and RNA was isolate using the RNeasy Mini Kit (Qiagen); and reverse transcribed using the QuantiTect Rev. Transcription Kit (Qiagen) according to the manufacturer’s protocol. RT-qPCR experiments were performed using TaqMan® Gene Expression Assays for arginase 1 and TaqMan® Gene Expression Master Mix (Applied Biosystems) on the Applied Biosystems StepOne^TM^ Real-time PCR system. RT-qPCR data was analysed via the comparative Ct method, using GAPDH as a house keeping control gene.

**Peritoneal macrophage gentamicin protection assay.** 2 x 10^5^ thioglycollate-elicited peritoneal macrophages were plated over-night onto tissue culture plastic in serum free OptiMEM media without antibiotics. Next morning, cells were washed 3 times with PBS and infected with 20 MOI of *Salmonella* Typhimurium M525. One hour after infection media was removed and cells were washed and incubated with 50 IU/ml gentamicin for another hour. At the end of gentamicin treatment, cells were washed in PBS and incubated in OptiMEM for further 5 hours without any antibiotics. At the end of the assay cells were lysed in 1% Triton, serial dilutions were prepared and l of each dilution were spotted into Ampicilin plates and colony forming units (CFU) were counted next day to calculate number of CFUs per ml.

**Stastistical analysis:** Where not already stated in subsections of text S1**,** for experiments comparing two groups, a Student T test was performed. For data with more than two groups, statistical analysis was performed using a nonparametric one-way analysis of variance (ANOVA) with Dunn‘s multiple comparison post-hoc test. For comparisons of groups with two or more factors, analysis was performed by two way ANOVA with Bonferroni multiple comparison post-test. Values of p<0.05 was taken to be significant in all cases. All tests were performed using the graphing and statistical software GraphPad Prism 5 (GraphPad Software, Inc, USA).

**Supplemental References**

1. **Skarnes WC, Rosen B, West AP, Koutsourakis M, Bushell W, Iyer V, Mujica AO, Thomas M, Harrow J, Cox T, Jackson D, Severin J, Biggs P, Fu J, Nefedov M, de Jong PJ, Stewart AF, Bradley A.** 2011. A conditional knockout resource for the genome-wide study of mouse gene function. *Nature* **474:**337-342.

2. **Pettitt SJ, Liang Q, Rairdan XY, Moran JL, Prosser HM, Beier DR, Lloyd KC, Bradley A, Skarnes WC.** 2009. Agouti C57BL/6N embryonic stem cells for mouse genetic resources. *Nat Methods* **6:**493-495.

3. **Hall LJ, Clare S, Dougan G.** 2010. NK cells influence both innate and adaptive immune responses after mucosal immunization with antigen and mucosal adjuvant. *J Immunol* **184:**4327-4337.

4. **Harrison JA, Villarreal-Ramos B, Mastroeni P, Demarco de Hormaeche R, Hormaeche CE.** 1997. Correlates of protection induced by live Aro- Salmonella typhimurium vaccines in the murine typhoid model. *Immunology* **90:**618-625.

5. **Beckonert O, Keun HC, Ebbels TM, Bundy J, Holmes E, Lindon JC, Nicholson JK.** 2007. Metabolic profiling, metabolomic and metabonomic procedures for NMR spectroscopy of urine, plasma, serum and tissue extracts. *Nature protocols* **2:**2692-2703.

6. **Ghosh S, Sengupta A, Sharma S, Sonawat HM.** 2012. Metabolic fingerprints of serum, brain, and liver are distinct for mice with cerebral and noncerebral malaria: a (1)H NMR spectroscopy-based metabonomic study. *Journal of Proteome Research* **11:**4992-5004.

7. **Lindon JC, Nicholson JK, Holmes E, Everett JR.** 2000. Metabonomics: metabolic processes studied by NMR spectroscopy of biofluids. *Concepts in Magnetic Resonance* **12:**289-320.

8. **Salek RM, Colebrooke RE, Macintosh R, Lynch PJ, Sweatman BC, Emson PC, Griffin JL.** 2008. A metabolomic study of brain tissues from aged mice with low expression of the vesicular monoamine transporter 2 (VMAT2) gene. *Neurochem Res* **33:**292-300.
