# Supplementary figures and images for: Infection Susceptibility in Gastric Intrinsic Factor (Vitamin B12)-Defective Mice Is Subject to Maternal Influences
Source: mBio. 2016 Jun 21;7(3):e00830-16. doi: 10.1128/mBio.00830-16 (PMC4916386; doi:10.1128/mBio.00830-16)

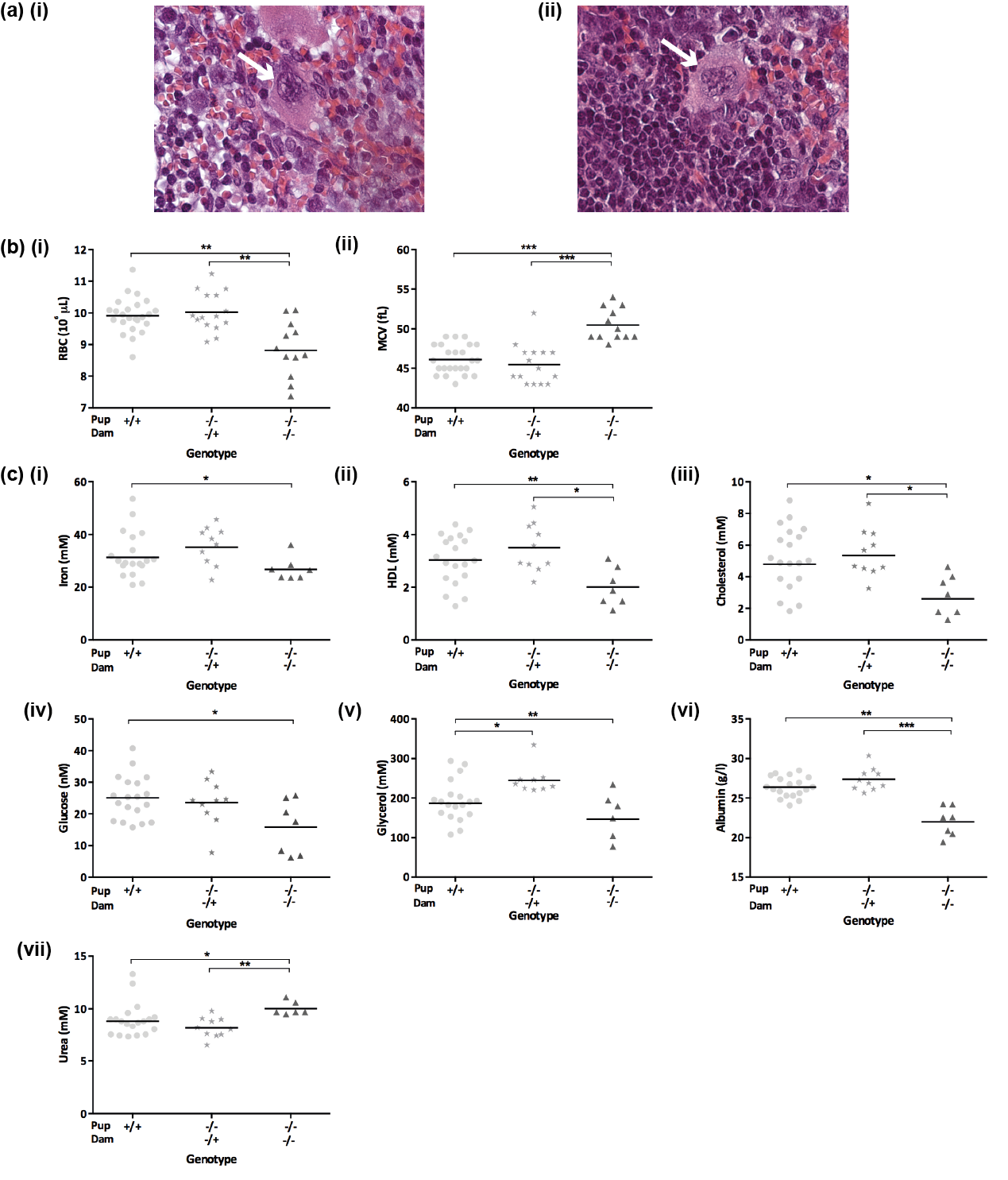

Supplement: Figure S1 — Histological, hematologic, and plasma defects are present in F2 Giftm1a/tm1a mice. (a) Representative hematoxylin and eosin stained spleen sections from naive F2 giftm1a/tm1a and wild-type mice showing that megakaryocytes were often hyperlobulated and hypersegmented in F2 Giftm1a/tm1a mice. Part i, wild-type mice; part ii, F2 Giftm1a/tm1a mice (×400 magnification). (b and c) Hematology and plasma chemistry analyses of F2 (black triangles), F1 (gray stars) Giftm1a/tm1a, and wild-type (gray circles) mice at 16 weeks of age. (a, part i) Total red blood cell count per microliter, (ii) MCV of red blood cells in femtoliters. (b) Concentrations of iron (millimolar) (i), high density lipoproteins (millimolar) (ii), cholesterol (millimolar) (iii), glucose (nanomolar) (iv), glycerol (nanomolar) (v), albumin (grams per liter) (vi), and urea (nanomolar) (vi) in plasma. Black bars represent geometric mean values. ***, P < 0.001; **, P < 0.01; *, P < 0.05 (ANOVA with Dunn’s multiple-comparison post hoc test). Download [file mbo003162863sf1.docx]

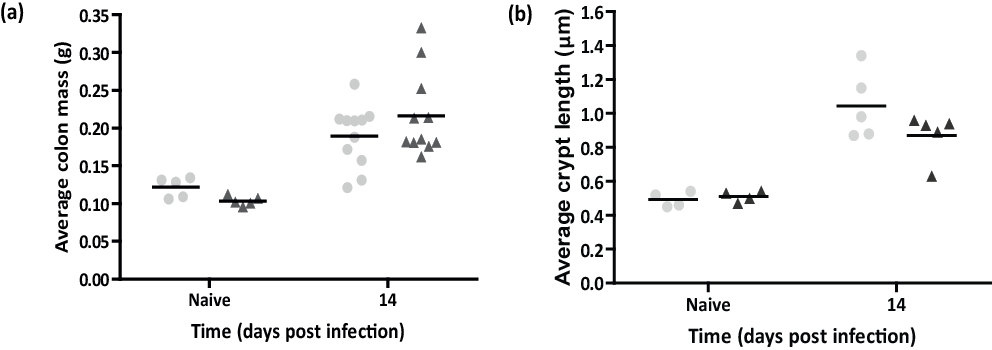

Supplement: Figure S2 — (a) Colon mass (grams) and (b) average colonic crypt length (micrometers) of naive and C. rodentium-infected (day 14 p.i.) F2 Giftm1a/tm1a and wild-type mice. Gray circles represent wild-type mice, and black triangles represent F2 Giftm1a/tm1a mice. Black bars represent geometric mean values. Download [file mbo003162863sf2.docx]

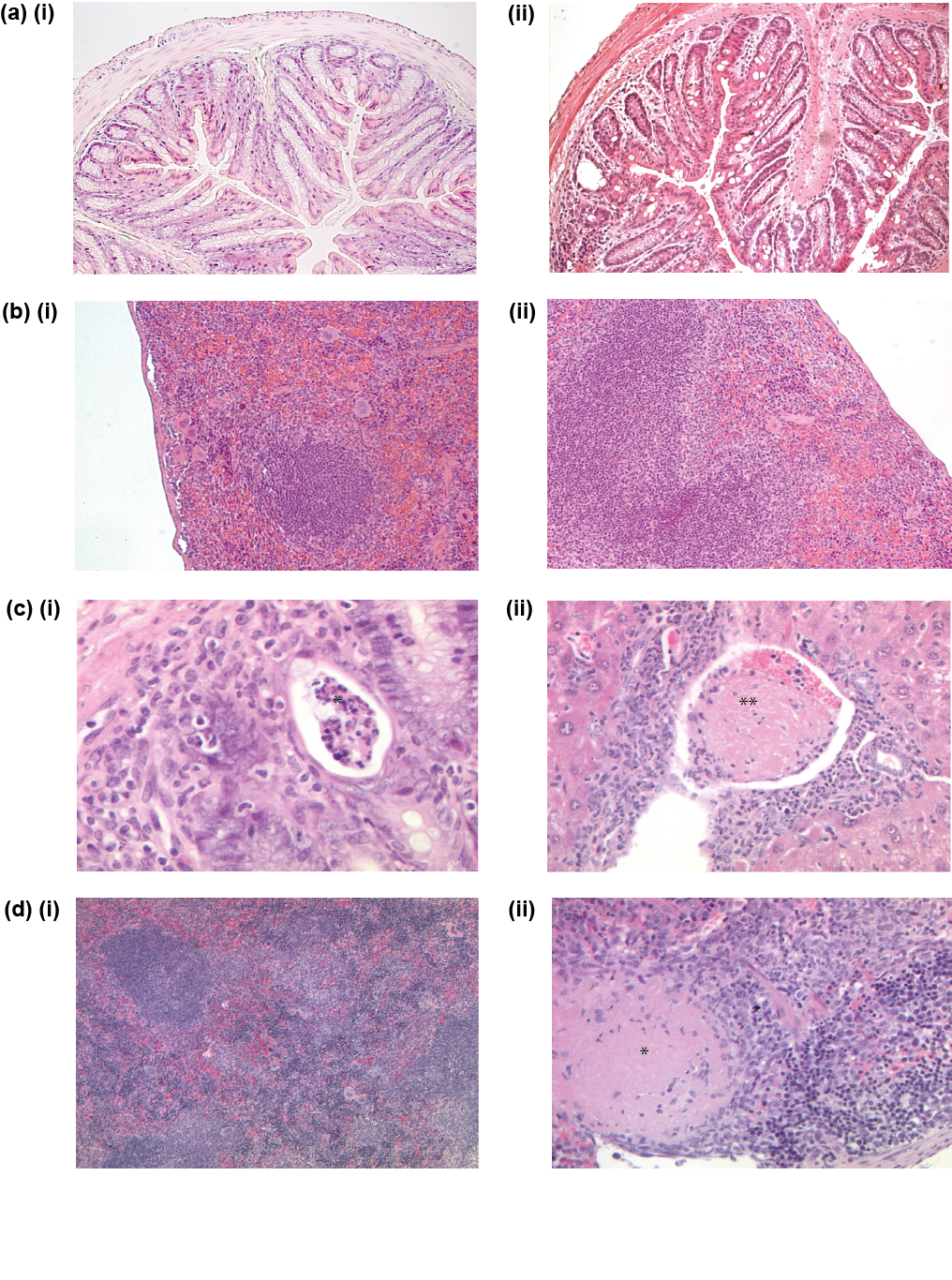

Supplement: Figure S3 — Representative hematoxylin- and eosin-stained sections from naive and infected F2 Giftm1a/tm1a and wild-type mice. (a) Naive wild-type (i) and Giftm1a/tm1a (ii) mouse colon tissues (×100 magnification). (b) Naive wild-type (i) and F2 Giftm1a/tm1a (ii) mouse spleen tissues (×100 magnification). (c) F2 Giftm1a/tm1a mice 14 days after C. rodentium infection: i, colonic crypt abscess (*; ×400 magnification); ii, thrombi in the hepatic portal vein (**; ×200 magnification). This was not observed in the livers of wild-type mice (data not shown). (d) Spleen sections from wild-type mice (i) (×100 magnification) and F2 Giftm1a/tm1a mice (ii) on day 14 after S. Typhimurium infection. Asterisks indicate large necrotic areas not seen in wild-type infected mice (×200 magnification). Download [file mbo003162863sf3.docx]

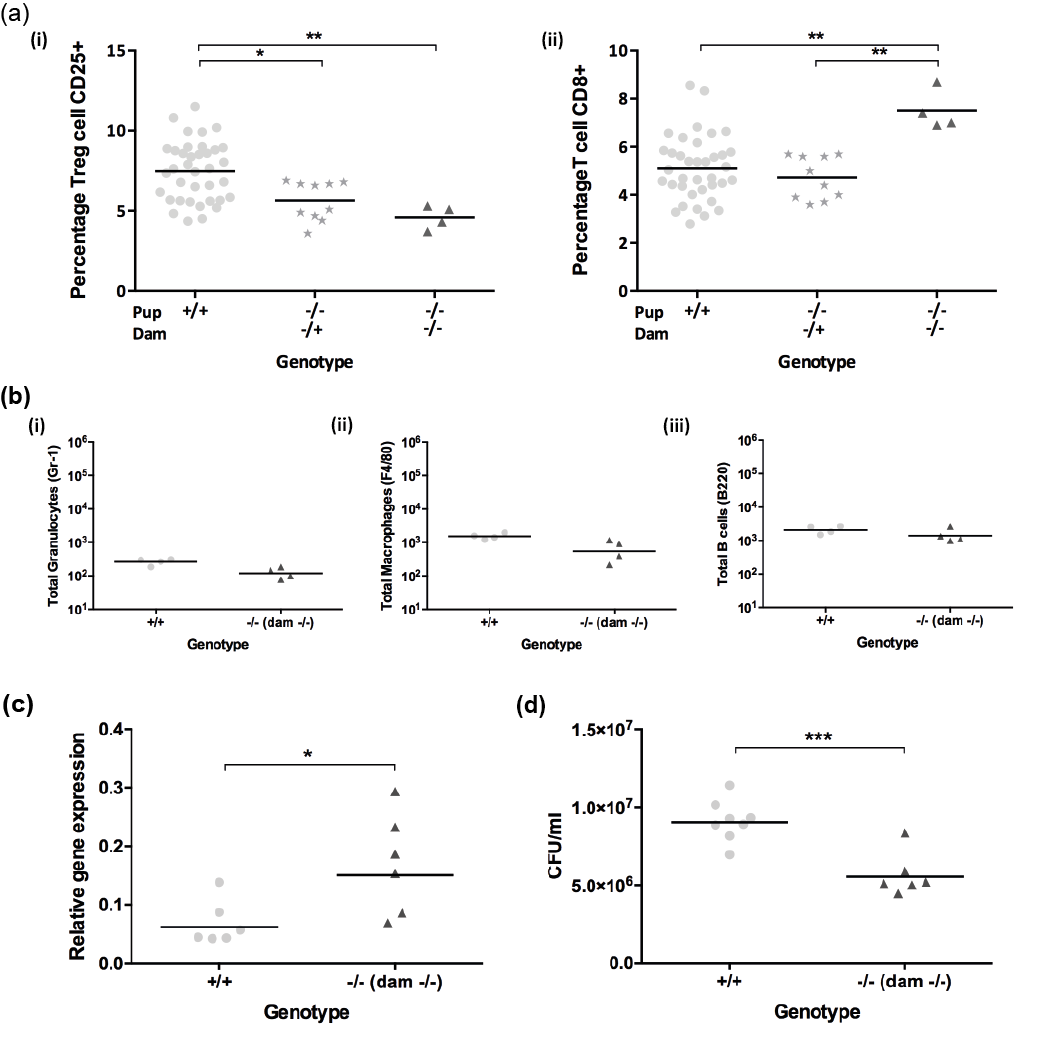

Supplement: Figure S4 — Profiling of immune cells from naive F1 Giftm1a/tm1a (gray stars), F2 Giftm1a/tm1a (black triangles), and wild-type (gray circles) mice. (a) Peripheral blood lymphocyte analysis of percentages of regulatory T cells (i) and CD8+ cytotoxic T cells (ii). All samples were analyzed on a BD LSR II analyzer. (b) Analysis of wild-type and Giftm1a/tm1a mouse splenocytes on day 14 after S. Typhimurium infection. Shown are the total Gr-1 (i), F4/80 (ii), and B220 (iii) cells. All samples were analyzed on a BD LSR Fortessa analyzer. Interpretation of the results was performed with FlowJo (v9). (c) Resident peritoneal macrophages were isolated from naive wild-type and F2 Giftm1a/tm1a mice, and relative expression of the arginase 1-encoding gene was compared by RT-qPCR. (d) The microbicidal abilities of wild-type and F2 Giftm1a/tm1a mouse macrophages were compared by gentamicin protection assay. Thioglycolate-elicited peritoneal macrophages were infected with S. Typhimurium M525 at an MOI of 20 in serum-free medium, and 5 h after gentamicin treatment, cells were lysed and plated on agar plates in different dilutions and CFU were counted. Black bars represent geometric mean values. Statistical analysis was performed by ANOVA with Dunn’s multiple-comparison post hoc test for panels a and b and Student’s t test for panels c and d. *, P < 0.05; **, P < 0.01; ***, P < 0.001. Download [file mbo003162863sf4.docx]

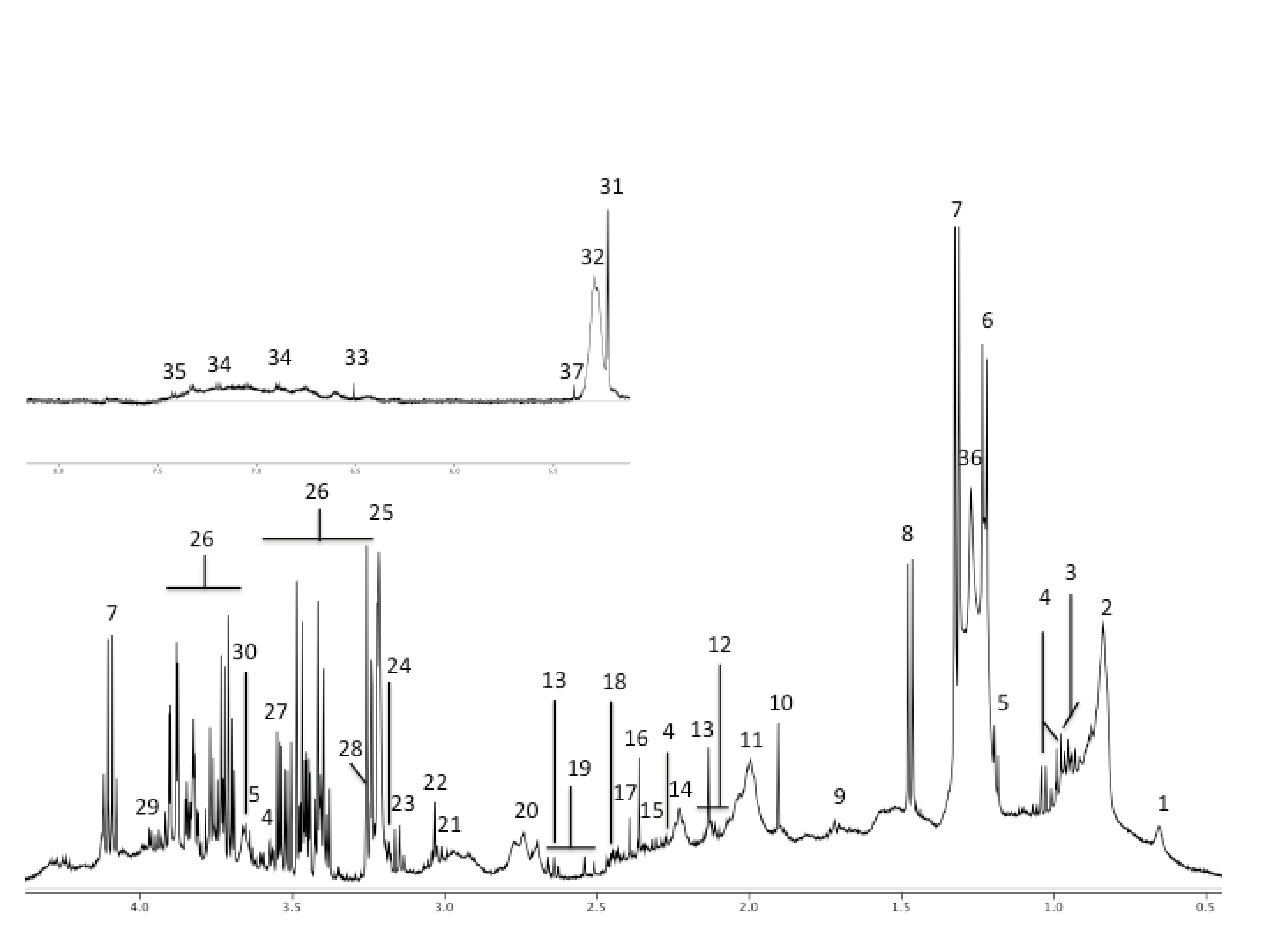

Supplement: Figure S5 — High-resolution 600-MHz 1H NMR spectrum of blood serum from F2 Giftm1a/tm1a mice. Peaks: mobile fatty acids and lipoproteins [1, −CH3/cholesterol; 2, −(CH2)n−; 36, −CH2CH2CO; 11, −CH2C═C; 14, −CH2C═O; 20, ═C-(CH2)–C═; 25, -N(CH3)3]; 3, leucine and isoleucine; 4, valine; 5, 3-hydroxybutyrate; 6, methylmalonate; 7, lactate; 8, alanine; 9, cadaverine-putrescine; 10, acetate; 12, glutamine and glutamate; 13, methionine; 15, 3-hydroxybutyrate; 16, pyruvate; 17, succinate and malate; 18, glutamine; 19, citrate; 21, lysine-cadaverine; 22, creatine; 23, methylmalonate; 24, choline; 26, glucose; 27, glycine; 28, betaine-taurine; 29, serine; 30, phenylacetylglycine; 31, alpha-glucose; 32, mobile unsaturated lipids; 33, fumarate; 34, tyrosine; 35, phenylalanine; 37, allantoin. Download [file mbo003162863sf5.docx]
